# Storage Profiling: Evaluating the Effect of Modified Atmosphere Packaging on Metabolomic Changes of Strawberries (*Fragaria × ananassa*)

**DOI:** 10.3390/metabo15050330

**Published:** 2025-05-15

**Authors:** Johannes Brockelt, Robin Dammann, Jennifer Griese, Agnes Weiss, Markus Fischer, Marina Creydt

**Affiliations:** 1 Hamburg School of Food Science, Institute of Food Chemistry, University of Hamburg, Grindelallee 117, 20146 Hamburg, Germany; johannes.brockelt@uni-hamburg.de (J.B.); robin.dammann@uni-hamburg.de (R.D.); markus.fischer@uni-hamburg.de (M.F.); 2 Hamburg School of Food Science, Food Microbiology, University of Hamburg, Ohnhorststrasse 18, 22609 Hamburg, Germany; jennifer.griese@uni-hamburg.de (J.G.); agnes.weiss@uni-hamburg.de (A.W.)

**Keywords:** food spoilage, FT-NIR, lipidomics, storage analysis, strawberry

## Abstract

**Background/Objectives:** Strawberries (*Fragaria × ananassa*) are among the most commonly consumed fruits due to their taste and nutritional benefits. However, their high rate of spoilage poses a major problem during the period from harvest and transport to further processing or marketing. The aim of this study was, therefore, to investigate the effects of passive modified atmosphere packaging on the metabolome and shelf life of strawberries as a more sustainable alternative compared to standard market storage conditions. **Methods:** A total of 99 strawberry samples were analyzed for microbial viable counts, water content, and metabolomic changes using non-targeted low-resolution near-infrared spectroscopy, high-resolution mass spectrometry, and microbial culture-based methods. **Results:** Using near-infrared spectroscopy as a rapid screening method, the linear regression model indicated that strawberries stored under modified atmosphere packaging conditions had a longer shelf life. Furthermore, lipidomic analysis using mass spectrometry showed that the levels of spoilage biomarkers, such as oxidized phosphatidylcholines and lysophosphatidylcholines, were increased under common market storage conditions without a controlled atmosphere. In contrast, the levels of these metabolites were reduced when strawberries were stored in modified atmosphere packaging. Moreover, the strawberries stored under modified atmosphere packaging had a lower number of bacteria, yeasts, and molds as well as a lower water loss throughout the entire storage period. **Conclusions:** Overall, the study highlights the potential of passive modified atmosphere packaging films to extend the shelf life and thus maintain the edibility of strawberries over a longer period.

## 1. Introduction

Strawberries are among the most widely consumed fruits and one of the most frequently cultivated berry fruits in the world. In addition to their sweet and fruity taste, strawberries are an important source of phytochemicals. In particular, their bioactive compounds, such as polyphenols, anthocyanins, and vitamins, are known for their antioxidant properties and thus for their positive effect on human health. Due to the high demand for this fruit, there is an increasing need to improve its shelf life. However, strawberries are highly perishable and, due to their fragile structure, susceptible to tissue damage during harvest, transport, and storage. Moreover, strawberries have a short shelf life, mainly because of their high respiration and metabolic rates during storage [1,2,3]. Furthermore, there is a significant loss of water during storage, which can generally lead to a loss of quality. Strawberries are susceptible to fungal infection, which can destroy entire batches. Various studies have identified particular molds such as *Botrytis cinerea* (gray mold), *Penicillium* spp., and *Rhizopus stolonifer* as the main cause of strawberry spoilage [4,5]. Therefore, it is important to understand and evaluate the processes that lead to these changes in the fruit in order to develop optimal conditions for improving the fruit’s shelf life after harvest. Various studies have described methods for extending the shelf life of food. Modified atmosphere packaging (MAP) is an option that has been increasingly developed in recent years to extend the shelf life of food and reduce perishability. MAP films consist of a perforated and therefore permeable packaging material that can be used to create a modified atmosphere inside the package. The atmosphere can be actively modified by introducing gas into the package, thereby displacing and replacing the original gases. This controlled enrichment involves introducing CO_2_, O_2_, N_2_, ozone, or mixtures of these gases into perforated packaging to slow down the ageing process of food. Additives such as menthol or cinnamaldehyde are also used to inhibit processes such as microbial spoilage [6,7]. In contrast, the passive modification of the atmosphere is based on gases that naturally diffuse through the film due to its permeability, combined with the respiration of food, to achieve the desired endogenous protective atmosphere. The perforation is designed to achieve the lowest possible O_2_ concentration while increasing the CO_2_ concentration. In addition, humidity and condensation can be controlled depending on the type of food being stored. As passive MAP does not require an external gas supply or additional processing steps, it represents a more sustainable alternative to active MAP by reducing resource consumption and energy use. Accordingly, the films and their properties are optimized to best suit the type of food stored concerning parameters such as water activity, breathability, and composition [8,9,10].

In the past, several studies have been conducted to investigate the storage processes of strawberries. For example, Shen et al. (2018) and Liu et al. (2019) reported on the analysis of stored strawberries using near-infrared spectroscopy (NIR) and high-performance liquid chromatography (HPLC) with an evaporative light-scattering detector (ELSD) [11,12]. These studies focused on fungal infection or sugar analysis, as well as shelf-life prediction models [11,12]. Mancini et al. (2020) also used NIR to examine stored strawberries for texture, acidity, color, and variety-specific differences [13]. In two studies, Simkova et al. (2023, 2024) concluded that quality-relevant ingredients such as ascorbic acid, sucrose, or the anthocyanin content partially decrease with different cold storage conditions and also when strawberries are thawed [14,15]. The influence of the weight and size of different strawberry varieties on these ingredients was also demonstrated [14,15]. Other studies with NIR have focused on parameters such as degree of ripeness, total polyphenol content, sensory shelf life, and determination of storage time [16,17,18]. Furthermore, it has also been shown in various studies that the controlled change of atmosphere in MAP film during strawberry storage inhibits bacterial growth, maintains the firmness of the strawberries, ensures changes in quality-relevant properties including hardness, moisture, vitamin C, total soluble solids (TSS), titratable acidity, and appearance and prolongs the overall shelf life of the strawberries [6,7,19,20,21,22,23,24,25]. In addition, high-resolution methods such as liquid-chromatography–mass spectrometry (LC-MS) or gas chromatography (GC-MS) have been used to analyze the quality of stored strawberries under different atmospheres [26,27].

The aim of this study was to obtain the most comprehensive analytical overview possible of the changes that occur in strawberries during storage in passive MAP film compared to the industry-standard storage conditions without special atmospheric conditions. To this end, a total of 99 strawberry samples were analyzed using a low-resolution, rapid FT-NIR spectroscopy approach to determine the shelf life of strawberries stored in MAP film relative to their expected postharvest storage time. In a further approach, high-resolution liquid chromatography–electrospray ionization–ion mobility-quadrupole time-of-flight–mass spectrometry (LC-ESI-IM-qTOF-MS) was used to detect the differences in lipid profiles. The lipidome analysis should reveal changes in the lipid composition during storage, as this is important for the stability of the cell membrane and thus has an impact on the sensory properties [28]. To enable a comprehensive assessment, other quality parameters such as water loss and microbial viable counts were also analyzed during storage.

## 2. Materials and Methods

### 2.1. Reagents and Chemicals

Demineralized water was ultra-purified using a Direct-Q 3 UV-R system (Merck, Millipore, Darmstadt, Germany). Acetonitrile (LC-MS grade), isopropanol (LC-MS grade), chloroform (HPLC grade), methanol (LC-MS grade), ammonium formate (LC-MS grade), sodium chloride (NaCl), plate count agar, and chloramphenicol were obtained from Carl Roth GmbH and Co. KG (Karlsruhe, Germany). Malt extract agar was purchased from VWR (Radnor, Pennsylvania, USA). The ESI tuning mix, hexamethoxyphosphazine, purine and hexakis(1H, 1H, 3H-tetrafluoropropoxy)phosphazine were obtained from Agilent Technologies (City of Santa Clara, CA, USA).

### 2.2. Sample Acquisition and Storage Design

The strawberry samples were harvested from a strawberry producer in the Hamburg area (Germany) on the day the trial began and were transported to the laboratories of the Hamburg School of Food Science at the University of Hamburg within 2 h. All the strawberries were of the Faith variety and were grown under conventional conditions in a polytunnel. Care was taken to ensure that the fruit was undamaged and free from visual signs of spoilage. The strawberries were divided at random into two batches and stored simultaneously under two different conditions in a KBS-E160 cooled incubator (Rubarth Apparate GmbH, Laatzen, Germany). The storage temperature was maintained at 4.0 ± 0.5 °C according to the specifications of the KBS-E160 refrigerated incubator. The strawberries were stored in closed containers without lighting. Relative humidity and air circulation were not actively monitored or controlled. For the control batch, the fruit was stored in open 500 g cardboard trays. These fruits represent the actual storage conditions that are common in retail trade. For the passive MAP storage batch, the strawberries were stored in perforated, heat-sealed Xgo preformed BOPP Xtend bags (StePacPPC, Tefen, Israel) with 8 laser perforations of 100 microns. The bags were sealed immediately after filling using a Caso VC 6 vacuum sealer (Caso Design, Arnsberg, Germany) in sealing-only mode without vacuum for 1.5 s. Nine samples were stored per storage day and storage condition, where one sample consisted of approx. 250–400 g of strawberries. The weight range of 250–400 g is based on the usual variation in practice as well as on the natural variation in size and shape of the strawberries. Each sample represented a different selection of strawberries, ensuring a representative dataset.

In order to analyze the metabolic and viable count changes during storage, 9 samples per storage condition were taken on days 2, 4, 6, 8, and 10 for further analysis. The 10-day storage period was chosen to provoke measurable microbial, physicochemical, and metabolic changes and to demonstrate potential differences between the two storage methods. Although this duration exceeds typical shelf life under commercial conditions, it served as a controlled stress scenario to evaluate the preservation potential of passive MAP storage. A longer storage period did not seem to be appropriate, as a large proportion of the strawberries already showed visible mold at this point, and the strawberries were therefore no longer suitable for consumption (see Section 3.1). In total, this procedure resulted in 45 control samples and 45 samples for MAP storage. In order to observe a change compared to the fresh strawberries, a further nine samples were analyzed on the first day of storage (day 0). Each sample was analyzed for microbial viable counts, water content, and FT-NIR directly on the respective day of storage. For the LC-MS analyses, a part of each sample was shock-frozen with liquid nitrogen at −196 °C, then homogenized and freeze-dried directly on the respective storage day. Afterwards, the freeze-dried strawberries were stored at −80 °C until analysis (for the detailed sample preparation protocol for the LC-MS analysis, see Section 2.6.1). Table S1 summarizes the sample information with storage date, storage conditions, and sample ID.

### 2.3. Determination of the Viable Counts

For the microbial analyses of both storage conditions (MAP and control), the viable counts of aerobic mesophilic microorganisms, yeast, and mold were determined for each storage period. For this purpose, approx. 25 g of strawberries from three samples per storage condition and storage day were homogenized with 225 mL of 0.9% sterile NaCl solution each in a 400 mL homogenizing bag with a side filter (Carl Roth, Karlsruhe, Germany) using a Stomacher 400 Circulator laboratory mixer (Seward Laboratory, London, England) for 2 min at 250 rpm. Subsequently, a decimal dilution series (up to 10^−6^) was prepared from the homogenized sample solutions using 0.9% NaCl solution. For the aerobic mesophilic bacteria count, 100 µL of each dilution was spread-plated in duplicate on plate count agar and incubated at 36 °C for 72 h. To determine yeast and mold counts, the dilutions were plated in duplicate on malt extract agar with 0.2 mg/L chloramphenicol as an antibiotic and incubated at 30 °C for 72 h. The counts for aerobic mesophilic bacteria, yeasts, and molds were calculated and averaged across the three samples per condition and storage day.

### 2.4. Determination of the Water Content

In order to investigate the water content of the strawberries during storage, 15–25 g of each sample was dried on each storage day using a dry balance (SMART 6, CEM Corporation, Matthews, NC, USA) so that the water content of nine strawberry samples from the control storage and nine strawberry samples from the MAP storage could be determined per storage day. The water content of each sample was given as the mean value of nine samples per storage day and storage condition. The functionality of the dry balance corresponds to the water content determination of thermogravimetric measuring methods, such as sample drying in a drying cabinet.

### 2.5. Near-Infrared Spectroscopy Measurements

For the FT-NIR measurements on intact strawberry samples, three strawberries per strawberry sample were measured at 22 ± 2 °C on each storage day. Each strawberry was analyzed at three different locations on the fruit, resulting in nine technical replicates per sample. Thus, a total of nine strawberry samples, represented by three strawberries each, were analyzed by FT-NIR spectroscopy on each storage day per storage condition. The measurements were performed using a FT-NIR benchtop spectrometer from Bruker, equipped with an integration sphere and the OPUS 7.5 software (TANGO, Bruker Optics, Bremen, Germany). For each spectrum, 50 scans were recorded in a wavenumber range of 11,550–3950 cm^−1^ with a resolution of 4 cm^−1^.

The NIR spectra were pre-processed to ensure the comparability of the data. First, a multiplicative scatter correction (MSC) was applied to normalize the data. This step reduces effects such as additive and multiplicative scattering [29,30]. Afterwards, the data were binned to reduce the number of variables. For this purpose, five adjacent variables were averaged to one bin (binning (5)), resulting in a reduction from 3720 variables to 744 NIR wavenumber. The formation of bins does not necessarily lead to a loss of information, as in NIRS, neighboring wavenumbers are strongly correlated [31,32,33,34]. In the final step, the arithmetic average of the nine spectra of a sample was calculated. All data pre-processing was performed using the Unscrambler 11 software (Aspen Technology Inc., Bedford, MA, USA).

### 2.6. LC-ESI-IM-qTOF Measurements of Nonpolar Metabolites

#### 2.6.1. Sample Preparation

To extract the samples for LC-MS measurements, three to four strawberries per sample were divided in quarters with a ceramic knife and flash-frozen in liquid nitrogen at −196 °C. The frozen samples were homogenized in a knife mill (Retsch, Haan, Germany) with dry ice at a ratio of 1:1. Subsequently, the frozen homogenates were freeze-dried for three days (Beta 2-8 LSCplus, Martin Christ, Osterode am Harz, Germany). The freeze-dried strawberry powders were stored at −80 °C until further analysis. Nonpolar metabolites of the freeze-dried strawberries were extracted using a modified method based on the approach of Bligh and Dyer [35,36]. A total of 50 ± 0.5 mg of the freeze-dried strawberry powders were weighed into a 2 mL reaction tube (Eppendorf, Hamburg, Germany) and mixed with 0.75 mL of chloroform/methanol (1:2, *v*/*v*). Two steel balls (⌀ = 3.25 mm) were added, and the suspensions were ball-milled for 1 min at 3 m/s by using a Bead Ruptor 24 equipped with a 2.0 mL microtube carriage kit (Biolabproducts, Bebensee, Germany). Subsequently, 0.5 mL of ultra-purified water and 0.25 mL of chloroform were added and ball-milled again for 2 min at 3 m/s. The suspensions were then centrifuged for 20 min at 12,400× *g* and 4 °C (Eppendorf Centrifuge 5427 R, Eppendorf, Hamburg, Germany). Finally, the supernatants were diluted 1:4 (*v*/*v*) with chloroform and centrifuged again for 5 min. To prevent changes in the metabolites during extraction, the samples were kept on ice whenever possible, and cooled (4 ± 2 °C) solvents were used.

#### 2.6.2. LC-MS Conditions

Non-targeted lipidomic analysis was conducted using a 6560 Ion Mobility qToF LC-MS system (Agilent Technologies, Santa Clara, CA, USA). Liquid chromatography was performed using an Agilent 1290 Infinity II UHPLC system, which was equipped with a high-speed pump (G7120A, 1290 High Speed Pump, Agilent Technologies, Santa Clara, CA, USA), a multisampler (G7167B, 1290 Multisampler, Agilent Technologies, Santa Clara, CA, USA), and a temperature-controlled column tray (G7116B, 1290 MCT, Agilent Technologies, Santa Clara, CA, USA). Separation of metabolites was achieved with an Accucore RP-MS UPLC column (150 mm × 2.1 mm, 2.6 µm) equipped with a precolumn of the same material (10 mm × 2.1 mm, 2.6 µm) (Thermo Fisher Scientific, Braunschweig, Germany). Measurements were performed at a temperature of 50 °C and a flow rate of 0.3 mL/min. The mobile phase used was water (A) and acetonitrile/isopropanol (2:1, *v*/*v*) (B) with the addition of 0.1 mmol/L ammonium formate. Table S2 in the supporting information shows the gradient used for the LC-MS measurements of the strawberry extracts. The injection volume was set to 8 μL, and the autosampler was temperature controlled to 5 °C.

The measurements were executed in positive ionization mode within a mass range from 50 to 1700 Da, utilizing the following ionization parameters: gas temperature, 225 °C; drying gas flow rate, 10 L/min; nebulizer pressure, 40 psi; sheath gas temperature, 375 °C; sheath gas flow rate, 12 L/min; and capillary voltage, −3000 V. The following IMS parameters were employed: drift gas nitrogen; drift gas pressure, 3.95 Torr; frame rate, 0.9 frame/s; IM transient rate, 18 IM transients/frame; max. drift time, 60 ms; TOF transient rate, 600 transients/IM transients; trap fill time, 3900 μs; trap release time, 250 μs; and multiplexing pulse sequence length, 4 bits. Drift times were calibrated by infusing the ESI tuning mix and hexamethoxyphosphazine under the same measurement conditions as the samples once a day for a duration of one minute.

Prior to the series of measurements, the instrument was calibrated with ESI tuning mix. Furthermore, a lock mass calibration was conducted during the measurements via a secondary sprayer with purine and hexakis(1H, 1H, 3H-tetrafluoropropoxy)phosphazine. To ensure reproducibility, a quality control (QC) sample was injected after every ten measurements. The QC sample was prepared by mixing 10 µL of each extract. Additionally, a blank sample, which involved no injection, was analyzed every five measurements. Samples were measured in a random sequence to minimize potential instrumental drifts that could influence the results. To identify the most relevant compounds undergoing changes during storage, MS/MS fragment spectra were recorded at 10, 20, and 40 eV in QTOF mode. The acquisition rate/time for MS mode was 1.5 spectra/s with 666.7 ms/spectrum (transients/spectrum: 5452) and for MS/MS mode, 1 spectrum/s with 1000 ms/spectrum (transients/spectrum: 7999).

#### 2.6.3. LC-MS Data Processing

The multiplexing of IM-TOF data files was carried out using the PNNL Preprocessor (version 2020.03.23) with the following specific parameters: demultiplexing checked; moving average window size five frame; moving average smoothing checked; *m*/*z* not used; drift three; chromatography/infusion three; and signal intensity lower threshold 20 counts. Calibration of CCS values was performed with IM-MS Browser software (version 10.0). Four-dimensional feature finding was performed with Mass Profiler software (version 10.0) with the following parameters: restrict RT to 0–28.0 min for nonpolar measurement; ion intensity > 150.0 counts; isotope model common organic (no halogens); limit charge states to a range of 1 to 2; report single-ion features with charge state z = 1; retention time tolerance = ±10.0% + 0.50 min; drift time tolerance = ±1.5%; mass tolerance = ±20.0 ppm + 2.0 mDa; Q-score > 70.0. The bucket table was exported as .xls format. A bucket had to be detectable in at least 10% of all samples from one sample group, leaving 1661 variables for statistical analyses. A sum normalization and an autoscaling were performed on the dataset. The identification of lipids by MS/MS measurements was based on the high-resolution mass and fragment spectra and partially supported by the Lipid Annotator software as well as the Agilent Masshunter Qualitative Analysis 10.0 Software (Agilent Technologies) and the LipidMaps database [37]. In addition, CCS values were compared with the LipidCCS database [38].

### 2.7. Multivariate Data Analysis

Relevant variables were extracted using Analysis of Variance (ANOVA, *p*-value < 0.01) with false discovery rates (FDRs) according to Fisher’s Least Significant Difference (LSD) and Principal Component Analysis (PCA) as an unsupervised approach using MetaboAnalyst 6.0 [39]. In addition, a hierarchical cluster analysis using Euclidean distances and mean values was applied to the normalized and auto-scaled peak intensities of the identified metabolites with the MetaboAnalyst 6.0 software, as well [39]. Furthermore, regression models were built using rational quadratic gaussian progression regression (GPR) with MATLAB R2021b (The MathWorks Inc., Natick, MA, USA). For this purpose, a 3-fold cross-validation with 100 repetitions was conducted.

## 3. Results and Discussion

### 3.1. Changes in Viable Counts and Water Content During Storage

**Figure 1:**
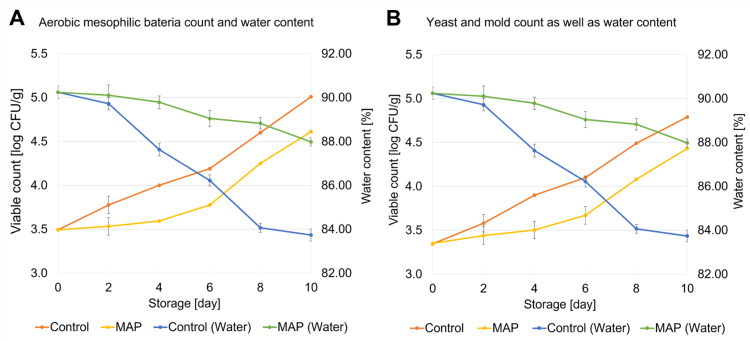
Plot of the average viable counts and standard deviations [log CFU/g] in relation to the water content and storage period from day 0 to day 10 of the aerobic mesophilic bacteria count (**A**) and the yeast and mold count (**B**). The viable counts of the control are shown in orange, and the ones of the MAP storage are shown in yellow; while the water content of the control storage is shown in blue, and the water content of the MAP storage is shown in green.

In order to investigate the influence of the MAP film on the microbial load of strawberries during storage, the number of aerobic mesophilic bacteria and the counts of yeast and mold were determined for each storage day and storage condition. The results of the total aerobic mesophilic bacterial count as well as the yeast and mold count for the control storage and storage in MAP film are shown in Figure 1 as well as in Tables S3 and S4 in the supporting information. The results of the water content for each storage day and storage condition are shown in Figure 1A,B as a green curve for MAP storage and a blue curve for control storage. Detailed water determination results are listed in Table S5 in the supporting information. Based on the control storage (orange curve in Figure 1A), the initial total aerobic mesophilic bacteria count in the fresh strawberries (day 0) was 3.5 log CFU/g. While this value increased minimally during the first four days, there was a significant rise in the aerobic mesophilic bacteria count to 5.1 log CFU/g from day 6 to day 10 of storage. A similar trend was observed for yeast and mold during control storage (orange curve in Figure 1B). On day 0, the count of yeast and mold was 3.4 log CFU/g. During storage, the count increased to 5.0 log CFU/g after 10 days of storage. The increase in the microbial load of strawberries during control storage is related to their perishability. Strawberries have a high respiration rate, which leads to moisture loss and creates a microclimate that favors the growth of microorganisms. Together with the relatively high water and sugar content, this accelerates the spoilage of strawberries [40]. In contrast, the count of aerobic mesophilic bacteria increased during storage in MAP film from 3.5 log CFU/g (day 0) to 4.8 log CFU/g (day 10) (yellow curve in Figure 1A). Similarly, the count of yeast and mold (yellow curve in Figure 1B) increased from 3.4 log CFU/g to 4.6 log CFU/g. In comparison to control storage, this indicates that passive MAP storage slows down the microbial growth by passively modifying the atmosphere during storage. The respiration of strawberries during storage consumes oxygen and produces carbon dioxide. In a semi-permeable system such as the MAP film, the O_2_ content can be reduced by the perforation, while the CO_2_ content accumulates, which can lead to a slowdown in bacterial growth [41,42,43]. Similar results were previously observed in a study, which showed that kiwifruit stored in a passive MAP film revealed inhibition of the bacterial growth [41]. Currently, there are no legally defined microbiological criteria in the European Union for total aerobic bacteria counts or for yeasts and molds in fresh, unprocessed strawberries (Regulation (EC) No. 2073/2005) [44]. However, several studies indicate that microbial loads above 4.5–6 log CFU/g can be associated with declining product quality and shelf life. For example, Wang et al. (2018) observed aerobic bacteria counts above ~4.5–5 log CFU/g in strawberries stored at 4 °C after extended storage, which correlated with a loss of firmness and overall quality [45]. Siro et al. (2006) reported visible mold growth and bacteria counts of up to 4.5–5.6 log CFU/g in air-stored strawberries at 7 °C [46]. In accordance with the other studies, the control samples in this study showed values above 5 log CFU/g for both aerobic mesophilic bacteria and yeast/molds on day 10. These values were also associated with visible changes in individual strawberries, such as softening, unpleasant odor, and localized mold growth.

Furthermore, the changes in water content during storage for the control-stored strawberries (blue curve) and for the MAP-stored strawberries (green curve) in Figure 1 show that a decrease in water content was analyzed with increasing bacterial growth, particularly in the control storage. The strawberries lost 6.52% water within 10 days, starting from an initial water content of 90.24% on day 0 during control storage. It was particularly striking that the strawberries had already lost 2.55% of water after the fourth day of control storage. In comparison, a water loss of only 2.27% was observed by day ten during MAP storage. In general, transpiration of water through the surface of the strawberries into the ambient atmosphere takes place. In MAP storage, this can lead to a higher relative humidity in the atmosphere, which favors the transpiration of water from the surface of the strawberries and ultimately leads to less water loss. Overall, the results show that strawberries stored in MAP film lose less water, suggesting that the quality of the strawberries remains more stable during MAP storage [47,48,49].

### 3.2. NIR Spectroscopy of Strawberries

#### 3.2.1. Spectra Interpretation

**Figure 2:**
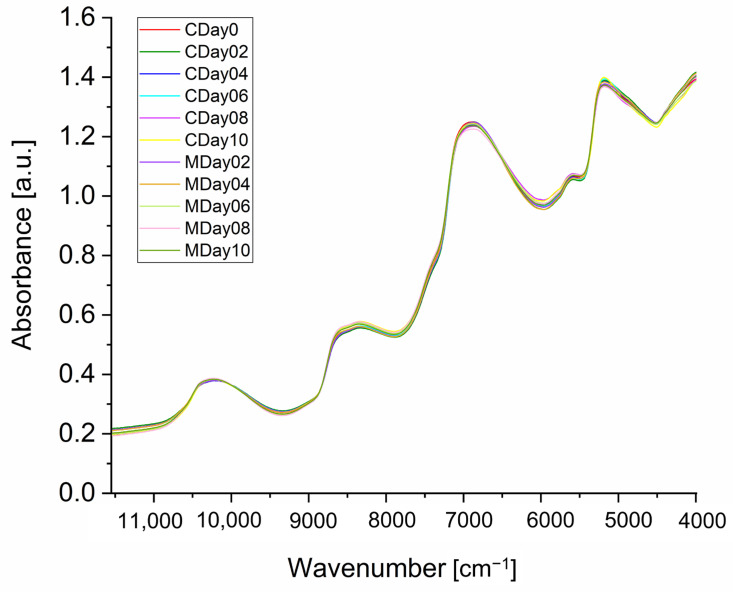
Averaged NIR spectra of all intact strawberries per storage day and storage condition (control (C) as well as MAP (M)) after MSC, binning of five adjacent variables, and mean centering.

FT-NIR spectroscopy was used as a rapid, albeit low-resolution, screening technique to analyze the differences between strawberries stored under the two conditions. Furthermore, a prediction model was to be developed to determine the shelf life of the strawberries that were stored in MAP film in relation to their predicted storage time. Strawberries consist of various nutrient classes such as water, carbohydrates, lipids, and proteins, which leads to overlapping peak signal intensities in the NIR spectra [50]. Therefore, it was not possible to assign the signal bands to molecules with complete accuracy. However, the bands could be assigned to the listed nutrient classes on the basis of different molecular oscillations and vibrations. Figure 2 shows the averaged FT-NIR spectra of all strawberries stored under control storage conditions (C) compared to those stored in MAP film (M).

The NIR spectra of strawberries showed absorption maxima in the range of 10,500–9500 cm^−1^. The bands in this region are attributed to the absorption of the second overtone of the O-H stretching vibrations and can be associated with water and carbohydrates. In addition, signals between 8800 cm^−1^ and 8200 cm^−1^ are due to the second overtone (HC = CH) of the C-H stretching vibrations of unsaturated fatty acids and by the second overtone of the C-H stretching vibrations. These regions can be associated with carbonaceous substances such as lipids. The absorbance bands between 7300 and 6000 cm^−1^ are characteristic of carbohydrates and proteins, resulting from the absorption the second overtone of the C-H bond and the first overtone of the O-H stretching vibrations, as well as the N-H stretching vibrations of proteins. The signal at ~5200 cm^−1^ is also caused by the combination of the O-H stretching vibration and the H-O-H bending vibrations in the first overtone region. These signals are primarily attributed to water but can also correlate with carbohydrates and proteins. The spectra also show significant absorptions at ~4500 cm^−1^ that can be attributed to the second overtone of the C-H stretching and bending vibrations of C-H_2_, associated with carbon–hydrogen absorption like lipids or carbohydrates [29,51]. The signals obtained from the strawberries were consistent with the components of strawberries described in the literature [52].

#### 3.2.2. Application of Multivariate Data Analysis

**Figure 3:**
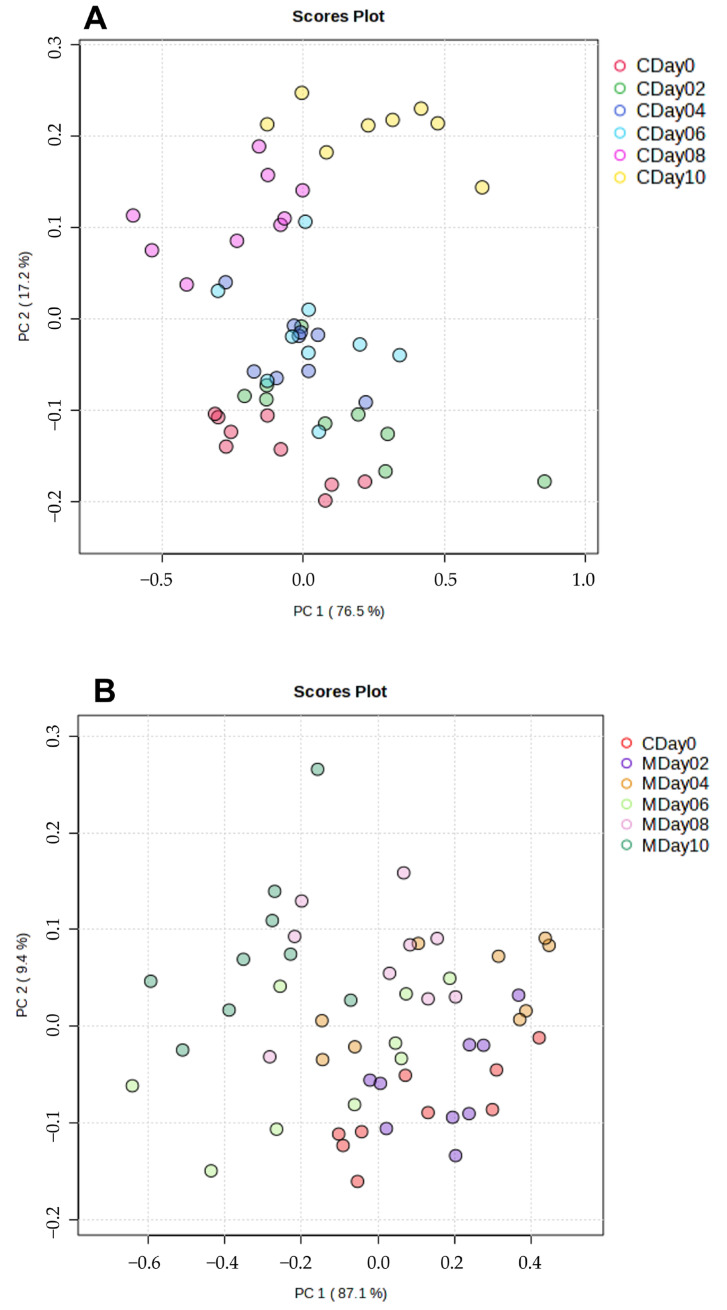
PCA score plots of PC1 and PC2 calculated using the FT-NIR spectroscopy. (**A**): PCA score plot of the control samples (C). (**B**): PCA score plot of the MAP samples (M).

In order to investigate differences between the storage conditions in relation to the storage days, the NIR datasets containing 744 variables were analyzed using PCA. Figure 3A,B show the PCA score plots of the first two principal components (PC1 and PC2) for the storage conditions compared to their storage days.

Analysis of the score plot (total variance 93.7%) of the control storage (Figure 3A) showed clear clusters according to the storage days. The samples from day 0 exhibit a negative correlation with PC2, while samples with increasing storage time show an increasingly positive correlation with PC2. It can be observed that samples from day 0 to day 6 are located in the negative range of PC2, whereas the samples from day 8 and day 10 cluster in the positive range of PC2. A similar result is seen in the score plot of the MAP storage (Figure 3B), which shows an overall variance of 96.5%. The score plot demonstrates that the samples from day 0 and day 2 cluster in the positive range of PC1, while the samples with increasing storage time (day 4 to day 10) show a distribution in the negative direction of PC1. The score plot of both conditions (Figure S1A, Supplementary Materials) illustrates that the samples from day 0 to day 6 of both storage conditions cluster in the negative range of PC2, while the samples from day 8 and day 10 cluster in the positive range. It can be observed that the samples cluster in the positive range of PC2 with increasing storage time. Furthermore, the score plot indicates that the control samples are further away from the samples of day 0 with increasing storage time (day 8 to day 10) than with storage under MAP. In summary, the PCA analysis of the FT-NIR spectroscopy analysis shows that the two storage conditions correlate with the storage time. Additionally, the score plot in Figure S1A shows differences between the two storage conditions.

**Figure 4:**
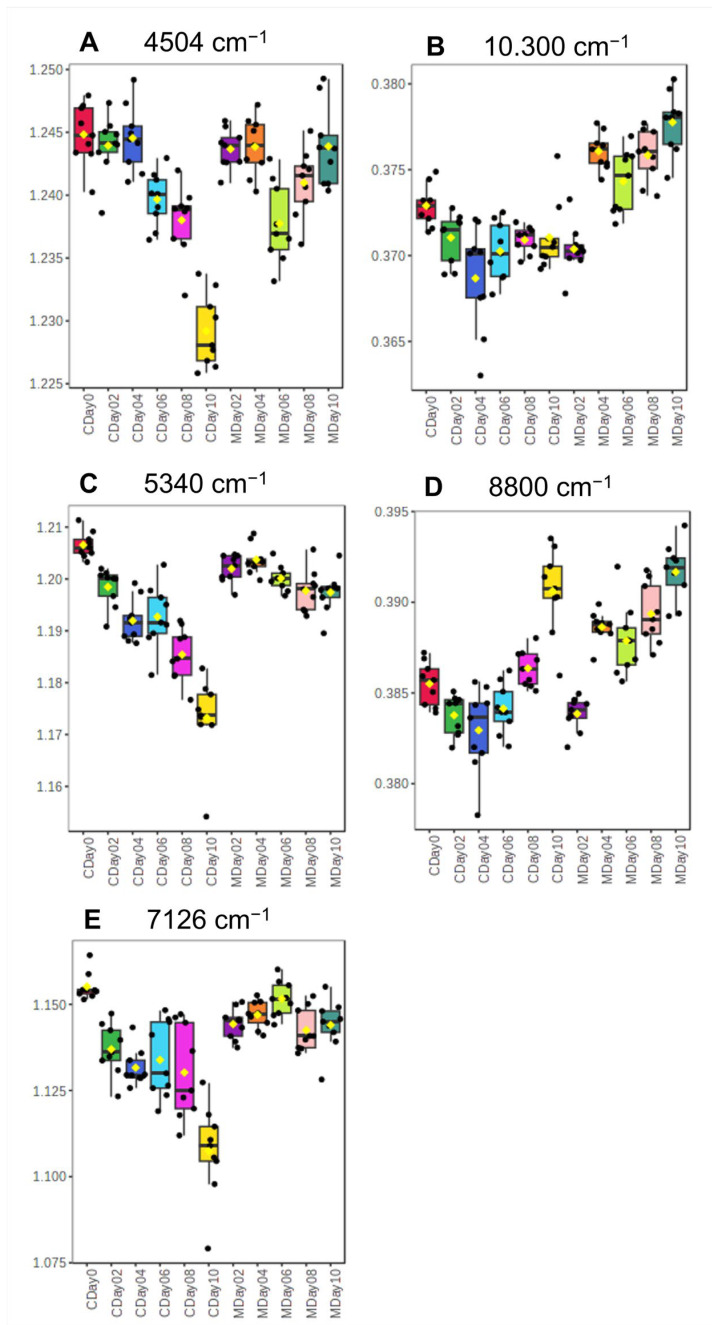
Boxplots of the most important selected wavenumbers recorded by FT-NIR and selected by ANOVA. Shown are the normalized signal intensities of the control samples (C) and the MAP samples (M) during the storage period of day 0 to day 10. (**A**) presents the boxplots of the signal intensities of the selected wavenumber 4504 cm^−1^ associated with carbohydrates, while (**B**) shows the boxplots of the signal intensities of the selected wavenumber 10.300 cm^−1^. (**C**) shows the boxplots of the signal intensities of the selected wavenumber 5340 cm^−1^, which is also associated with water content. (**D**) presents the boxplots of the signal intensities of the selected wavenumber 8800 cm^−1^, which is associated with lipids and (**E**) shows the boxplots of the signal intensities of the selected wavenumber 7126 cm^−1^, which is associated with proteins and carbohydrates.

To determine the relevant variables responsible for the differences between MAP and control storage, an ANOVA (*p*-value < 0.01) was applied to the NIR dataset. The results of the first 50 variables are summarized in Table S6 in the supporting information. The major wavenumbers in the regions of 5300 cm^−1^, 10,300 cm^−1^, 8800 cm^−1^, 4500 cm^−1^, and 7100 cm^−1^ were selected for analysis in order to identify differences between the storage conditions. Representative signal intensities of selected wavenumbers are shown as boxplots for both storage conditions in Figure 4. The variable at 4504 cm^−1^ (Figure 4A), which can be associated with carbohydrates, shows a decrease in intensity from day 0 to day 10 for the control storage. During control storage, strawberries were exposed to more O_2_ and increased respiratory activity, which led to an increased oxidative degradation of the carbohydrates [49]. The increase in the growth of bacteria, yeasts, and molds also triggered further degradation processes. Bacteria as well as yeast and mold use carbohydrates as an energy source, which can result in changes in the carbohydrate content of the fruit. The inhibition of bacterial growth during passive MAP storage thus indirectly leads to a inhibition in the carbohydrate degradation content [53,54]. Similar trends can be seen in the wavenumbers 5340 cm^−1^ and 10,330 cm^−1^ (Figure 4B,C), which are linked to water absorption. The signal intensities in these regions decrease with increasing storage time in the control storage, while they remain stable or even increase in the MAP storage (see Figure 4C). This may be due to the respiratory activity during control storage, which leads to a reduction in the water content. These results confirm the findings of the previous determination of the water content, as already explained in Section 3.2. The signal intensity of the wavenumber 8800 cm^−1^ (Figure 4D) shows an increase with prolonged storage time under both storage conditions. This wavenumber is associated with aliphatic C–H compounds such as lipids. Signals in the region of 7100 cm^−1^ (Figure 4E), associated with proteins and carbohydrates, show a significant decrease in the control storage samples from day 0 to day 10, while the MAP storage samples show a stable signal until day 10. An inhibition of protein-degrading processes, as could be caused by proteases, is unlikely due to the endogenous change in the atmosphere. Therefore, it can be assumed that the observed trend is due to a general inhibition of microbial growth by MAP, which in turn leads to an indirect inhibition of microbial proteases. Microbial enzymatic processes can also break down pectin and cellulose, which can lead to degradation of the cell wall by cellulases or pectinases and affect the firmness of the strawberry [53,54,55,56,57]. Overall, the analysis of the wavenumbers shows that there were more significant changes during control storage. By comparison, these changes in signal intensity in the MAP storage did not occur until the 6th or 8th storage day.

**Figure 5:**
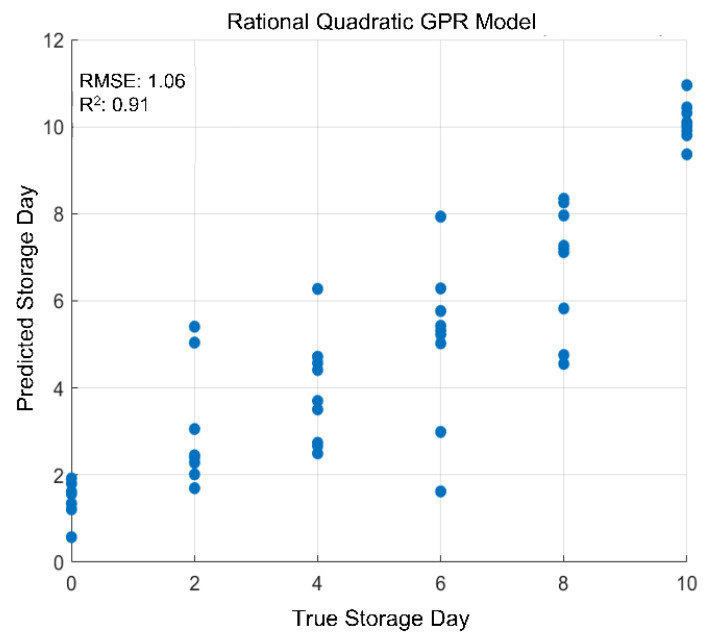
Linear regression model (Rational Quadratic GPR) of the strawberry samples stored in control storage to predict the storage day of the strawberry samples stored in MAP film. The blue dots show the prediction of the storage days depending on the actual storage day of the individual samples stored under MAP conditions.

A linear regression model was developed to determine the shelf life of strawberries stored in MAP film in relation to the predicted and actual storage days. To create the regression model, the NIR dataset was first divided into a training dataset and a test dataset. The NIR data from the control storage was used as the training dataset. For this purpose, the Rational Quadratic GPR algorithm with cross-validation (CV, 3-fold, 100 repeats) was applied to the training dataset. In addition, R^2^ and RMSE of this model were also determined. Subsequently, the NIR dataset of the MAP samples was used as a test dataset with this model, enabling the prediction of the shelf life of the strawberry storage in MAP film. The linear regression in Figure 5 showed an R^2^ value of 0.91 with an RMSE of 1.06%, indicating the efficacy of the GPR model in predicting the storage days of the control samples. The predicted storage days of the test set (MAP samples) and their actual values are summarized in Table 1.

The results presented in Table 1 show that there is no discernible impact of the MAP film at the beginning of the storage process on days 2 and 4. The samples were predicted (taking into account the standard deviation) to their true storage day. However, when looking at the predictions from day 6 to day 10, it is evident that the samples were predicted for a significantly earlier storage day than their true storage day. In terms of predicting the number of days Faith strawberries were stored in MAP film for 10 days, it can be concluded that MAP film resulted in an average increase in shelf life of 5 ± 2 days compared to industrial storage conditions. In order to make accurate predictions about the shelf life of the stored strawberries in the MAP film after harvest, care was taken to ensure that the strawberries used had the same degree of ripeness and were used directly from the field for the storage study. Nevertheless, the results of this study are limited in terms of different ripening levels and other varieties, which could also have an influence on shelf life after harvest and the storage of strawberries. Therefore, it is recommended that future studies analyze different ripening levels and fruit varieties.

The aim of MAP storage is to facilitate the passage of oxygen and water through the perforated film in rapidly perishable matrices such as strawberries, while allowing the diffusion of CO_2_ into the package, which accumulates accordingly [53,54]. These processes result in an endogenous protective atmosphere that reduces respiratory activity and minimizes chemical changes in the strawberries. In addition, by changing the atmosphere during storage, MAP film may inhibit microbial growth and thus indirectly reduce these enzymatic processes, which can preserve the quality and the shelf life of stored strawberries.

### 3.3. Lipidomic Profiling via LC-MS

**Figure 6:**
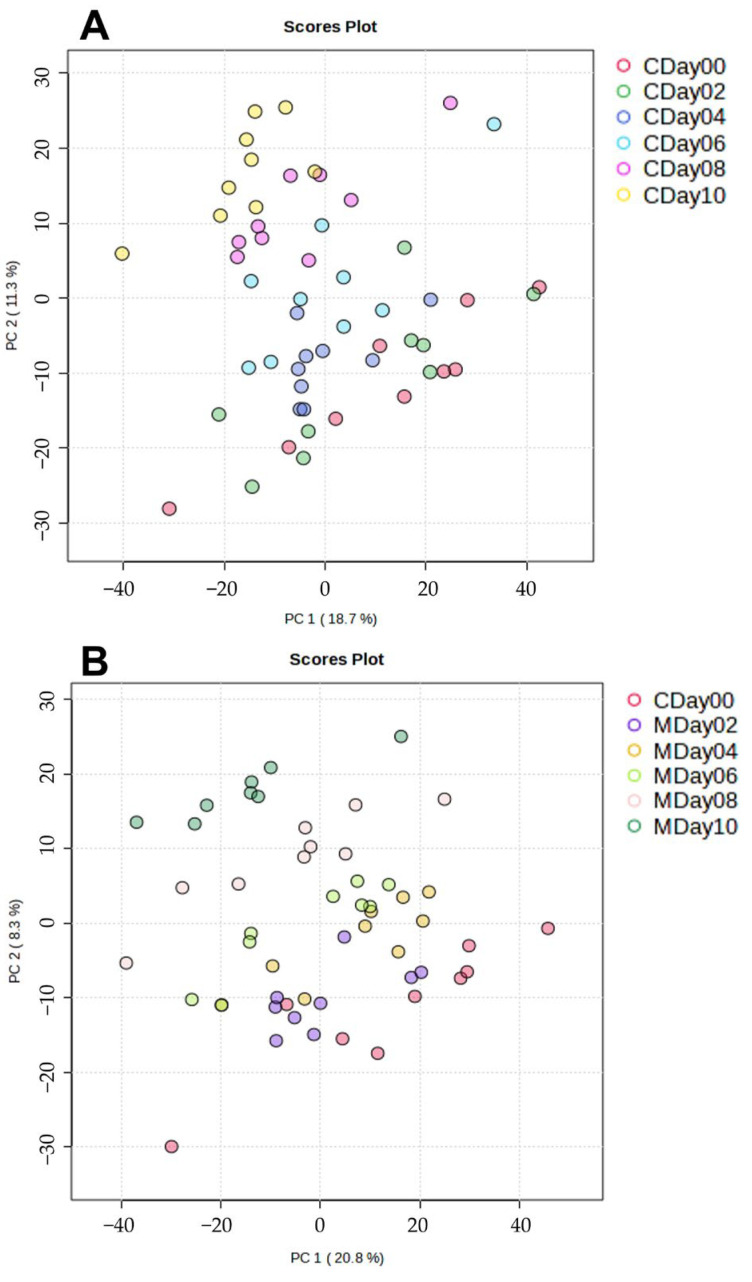
Score plots of the LC-MS analyses of the control strawberries (**A**) and the strawberries stored under MAP conditions (**B**).

To investigate the influence of both storage conditions on the lipidome composition, samples were analyzed using high-resolution LC-ESI-IMS-qTOF [53]. In contrast to the analysis of intact strawberries using FT-NIR spectroscopy as a rapid method, freeze-dried strawberries of each storage condition and storage day were analyzed. The first analysis of the differences was conducted using PCA of the peak intensities obtained for 1661 variables. Figure 6 depicts the score plots for PC1 and PC2 of the control storage (Figure 6A) and the MAP storage (Figure 6B). The control storage has a total variance of 27.2%, while the MAP storage exhibits a total variance of 30.0%. The PCA plot of the control sample clearly shows that the first days of storage (day 0 and day 2) form separate clusters in the positive area of the PC1 cluster. As the storage period progresses, the clusters of the individual storage days from day 4 to day 10 shift into the negative area of PC1 and simultaneously into the positive area of PC2. The clustering of the individual storage days can be observed. A similar trend can be seen in the score plot of the MAP samples. The strawberry samples from day 0 cluster in the positive part of PC1. With increasing storage time (day 2 to day 10), the samples show a clustering tendency in the positive direction of PC2. It is therefore apparent that samples of day 0 to day 4 are grouped into clusters. The total variance is 25.9% when the two storage conditions are analyzed together by PCA (Figure S1B, supporting information). The PCA plot also demonstrates that fresh strawberries in MAP storage on day 2 and day 4 clustered primarily with the fresh strawberries in control storage (day 0 and day 2) in the positive direction of PC2. However, with increasing storage duration, the samples from day 8 and day 10 of both conditions show clusters in the positive direction of PC1. In summary, the PCA plot of the LC-MS data reveals that there are differences between both storage conditions. With increasing storage time, the samples (day 6 to day 10) show a progressive shift away from the fresh strawberries (day 0). This trend occurred under both storage conditions.

**Figure 7:**
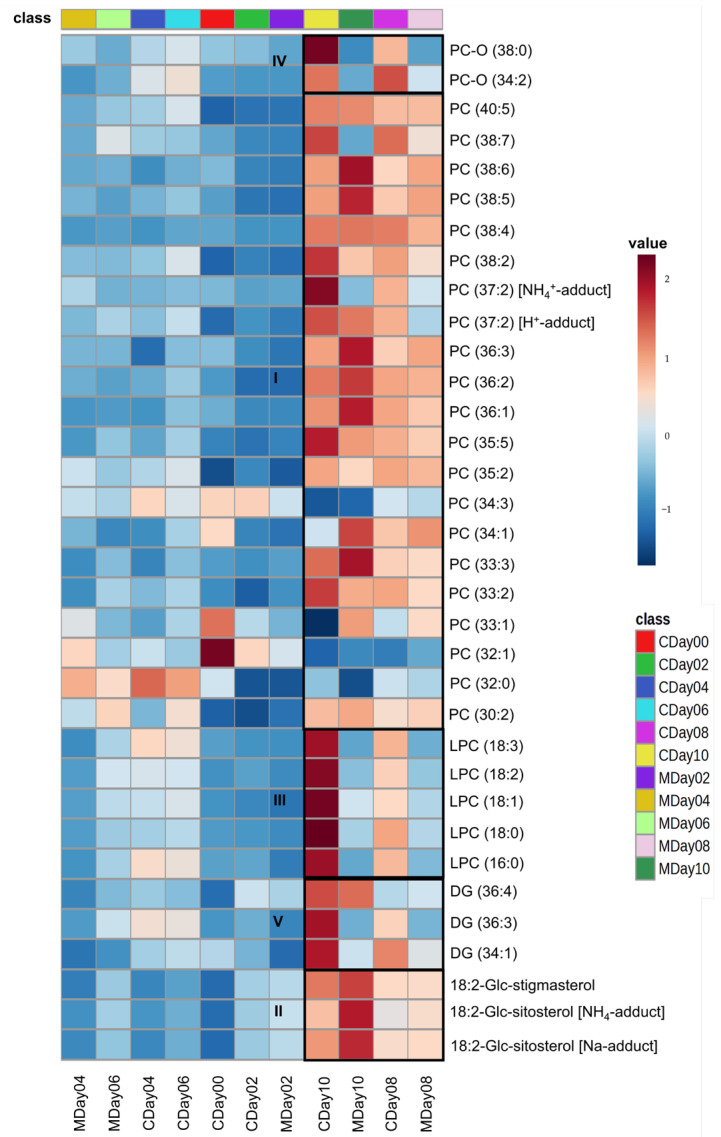
Results of the relation analysis of the identified metabolites. A hierarchical cluster analysis using Euclidean distances and the average was applied to the normalized and auto-scaled peak intensities. The intensity of the coloring indicates the peak intensity of the identified metabolite between the respective storage day and storage condition. The clusters are labeled with I–V and were assigned to: (I) identified PCs, (II) identified sterols, (III) identified LPCs, (IV) identified PC-Os, and (V) identified DGs.

**Figure 8:**
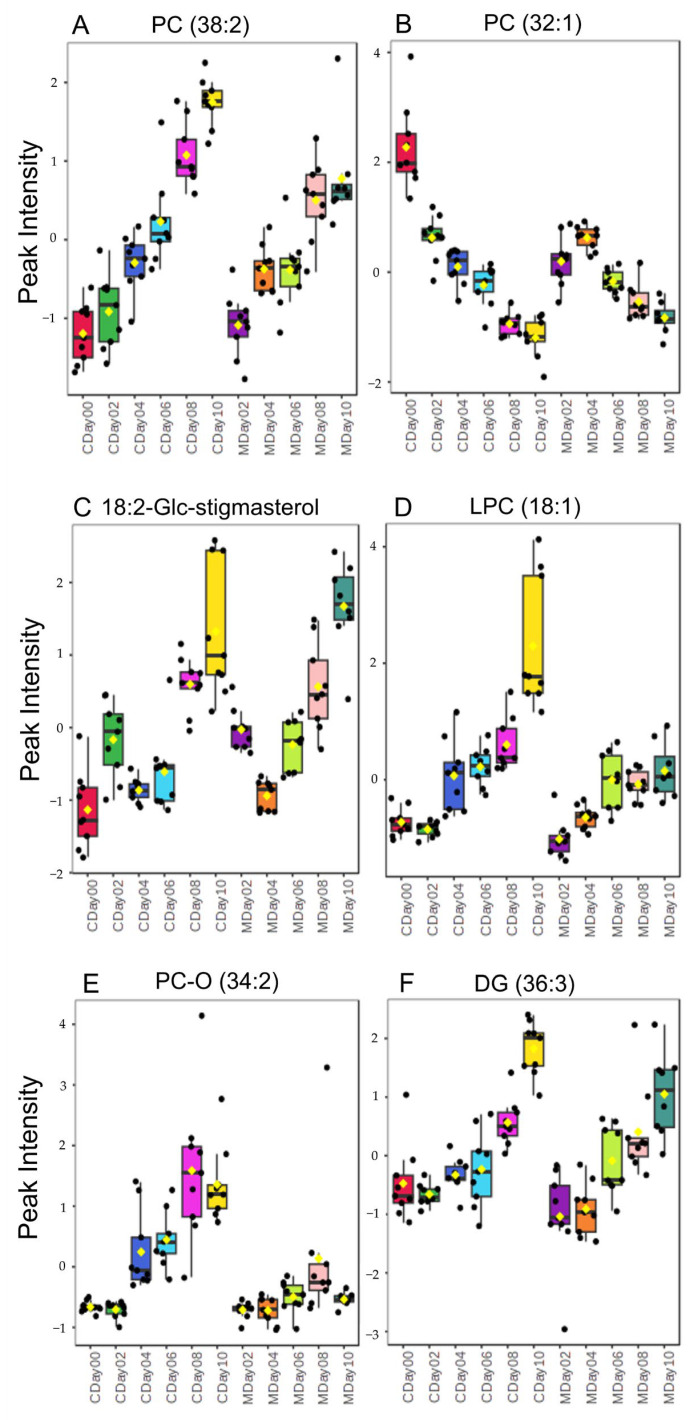
Boxplots of the normalized and autoscaled signal intensity of (**A**) PC (38:2), *m*/*z* 814.6292; (**B**) PC (32:1), *m*/*z* 732.5538; (**C**) 18:2-Glc-stigmasterol, *m*/*z* 854.6856; (**D**) LPC (18:1), *m*/*z* 522.3533; (**E**) PC-O (34:2), *m*/*z* 774.5613; and (**F**) DG (36:3), *m*/*z* 601.5164; which were chosen exemplarily from the selected metabolites for the differentiation of the two storage conditions (control (C) and MAP (M) storage).

To identify metabolites that have a significant impact on the quality of strawberries stored under both conditions, an ANOVA with a *p*-value < 0.01 was applied to the LC-MS dataset. A total of 34 variables showing differences during the storage period were identified on the basis of their MS/MS spectra. The majority of the identified lipids were phosphatidylcholines (PC) (in total 28) including lyso-PCs (LPC) as well as oxidized PCs (PC-O), which exhibited ionization as [M + H]^+^ or [M + NH_4_]^+^ adducts. PCs were identified by the fragment resulting from the cleavage of the phosphocholine fragment at *m/z* 184.07 and the calculated exact mass. Furthermore, the measured and calculated CCS values were employed as identification parameters. Additionally, two sterols and three diacylglycerides (DGs) were identified based on the fragmentation patterns of fatty acid cleavage and neutral loss of fatty acids [58,59]. The comprehensive list of all identified molecules with associated identification parameters is provided in Table S7, and representative MS/MS spectra of the identified lipids are presented in Figures S2–S4 in the supporting information. The heatmap in Figure 7 shows the relationships between the normalized and auto-scaled peak intensities of the identified metabolites and the storage days as well as the storage conditions. Figure 8 shows a selection of boxplots of the normalized peak intensities of the identified lipids.

The heatmap provides different types of clusters depending on the identified substance class and storage date. The largest cluster is Cluster I in Figure 7, which represents the identified PCs. It can be seen that the highest intensities for most of the identified PCs occur with increasing storage duration. This is exemplarily shown in the boxplots of PC (38:2), *m*/*z* 814.6292, in Figure 8A. The results indicate that there is an increased synthesis of phospholipids in the plant cells. The increase of some phospholipids during storage of strawberries can be explained by stress reactions of the cell membranes. Phospholipids are essential building blocks of cell membranes and fulfill various protective functions. In situations that pose an increased stress factor, such as storage at 4 °C (cold stress), phospholipids can be synthesized to a greater extent. Similar results have already been found in various studies regarding the cold storage of pineapples and peaches. These studies described an increased phospholipid content or a reduced degradation during cold storage as a stress-induced reaction to strengthen the cell membrane and reduce oxidative stress [60,61]. This aspect could also explain the increase in intensities at wavenumber 8800 cm^−1^ from the FT-NIR spectral analysis (Figure 4D), which correlate with the lipid content and also increase with progressing storage time in both storage conditions. The increase in PC could also be due to changes in viable counts. This could be confirmed by the fact that the course of the PC increase correlates with the increase in microbial growth during storage. While PC synthesis occurs in about 10% of all bacterial species, it is more typical for eukaryotes such as yeasts and molds. This suggests that the observed PC accumulation during storage might be partially attributed to these microorganisms or could be induced by cold stress factors [62,63]. Nevertheless, two PCs were identified (PC (32:1), *m*/*z* 732.5538 and PC (34:3), *m*/*z* 756.5528) that were equally degraded during the two storage conditions (Figure 8B). This degradation can be triggered by the activation of phospholipase A and phospholipase D, which is considered as an essential step in the hydrolysis of phospholipids induced by stress, such as in postharvest conditions. This process leads to the hydrolysis of phospholipids and the formation of free fatty acids [64,65,66,67].

The analysis of cluster II in Figure 7, which shows the identified sterols, reveals that they have high-intensity peaks on the eighth and tenth days of both storage periods. It can be observed that the sterols are synthesized with increasing storage time (Figure 8C). The synthesis of these sterols can be influenced by stress factors such as low temperatures. Indeed, an increase in both sitosterol and stigmasterol was observed in studies on apple and tomato storage [67,68].

The LPCs identified in Figure 7 in cluster III showed significantly higher peak intensities on day 8 and day 10, especially during controlled storage. This can also be seen in Figure 8D, which shows, for example, the formation of LPC 18:1 under both storage conditions. Various studies have shown that an increased LPC content in plants can be an indication of reduced cell membrane integrity. This can be caused by a loss of water in the cells or by the microbial and enzymatic degradation of pectin and lipids in the cell walls [57,69,70,71]. Since a larger increase in LPCs was observed in particular in the control storage, this may indicate that wound damage to the cell membrane and thus signs of quality impairment occur to a lesser extent in strawberries stored under MAP.

The identified oxidized PCs show high peak intensities at storage days 8 and 10 of the control storage (cluster IV in Figure 7). This leads to the conclusion that oxidized PCs are formed as a result of fat oxidation. A comparison with Figure 8E (PC-O (34:2), *m*/*z* 774.5613) shows that these are formed to a greater extent during control than MAP storage.

Cluster V in Figure 7 shows the identified DGs. It can be seen that increasingly higher peak intensities occur on storage days 8 and 10 of the control storage. In addition, Figure 8F shows that DG (36:3) forms with increasing storage duration under both storage conditions but is more pronounced on day 10 of control storage than that under MAP storage. The synthesis of DG can be attributed to the prior degradation of triacylglycerides (TAGs) to DGs by enzymatic lipolysis [72,73,74].

In conclusion, LC-MS analysis showed that both storage regimes led to increased PC synthesis during storage, probably due to the low temperatures. However, more markers for cell membrane damage indicative of lipid oxidation and lipolysis were also identified during control storage than during MAP storage with increasing storage duration. This suggests that MAP storage may reduce lipid oxidation, lipolysis, and cell membrane damage.

## 4. Conclusions

The results of this study suggest that MAP films and the resulting production of an endogenous protective atmosphere during storage can positively influence the shelf life of strawberries, particularly with regard to microbial load and water content. This was verified by a rapid, non-targeted FT-NIR spectroscopy method to determine the shelf life. In addition, the lipidomic approach using LC-MS showed that certain biomarkers for strawberry spoilage, such as oxidized PC or LPC, could be detected in higher concentrations under control storage and only to a small extent in MAP storage. The results of the study show that changes in the metabolome were observed with both types of storage. However, MAP storage significantly reduces these changes, so in our study, the quality and shelf life of the strawberries of the Faith variety were maintained over a longer period of 5 ± 2 days compared to that under industry-standard storage conditions.

## Figures and Tables

**Table 1:** Results of the linear regression model predicting the storage day of strawberries in MAP. Shown are the individual samples from MAP storage (MDay2–10) as well as the true and predicted storage days, compared to the samples that were not stored in the MAP packages.

Day 2	True	Prediction	Day 4	True	Prediction	Day 6	True	Prediction
MDay2-1	2	2	MDay4-1	4	2	MDay6–1	6	4
MDay2-2	2	2	MDay4-2	4	3	MDay6–2	6	3
MDay2-3	2	3	MDay4-3	4	3	MDay6–3	6	3
MDay2-4	2	3	MDay4-4	4	3	MDay6–4	6	4
MDay2-5	2	2	MDay4-5	4	4	MDay6–5	6	4
MDay2-6	2	2	MDay4-6	4	4	MDay6–6	6	4
MDay2-7	2	2	MDay4-7	4	2	MDay6–7	6	3
MDay2-8	2	1	MDay4-8	4	1	MDay6–8	6	8
MDay2-9	2	3	MDay4-9	4	4	MDay6–9	6	6
Mean value		2	Mean value		3	Mean value		4
σ		1	σ		1	σ		1
**Day 8**	**True**	**Prediction**	**Day 10**	**True**	**Prediction**			
MDay8-1	8	6	MDay10-1	10	5			
MDay8-2	8	5	MDay10-2	10	2			
MDay8-3	8	6	MDay10-3	10	5			
MDay8-4	8	6	MDay10-4	10	5			
MDay8-5	8	6	MDay10-5	10	8			
MDay8-6	8	6	MDay10-6	10	5			
MDay8-7	8	7	MDay10-7	10	7			
MDay8-8	8	2	MDay10-8	10	5			
MDay8-9	8	3	MDay10-9	10	4			
Mean value		5	Mean value		5			
σ		1	σ		2			

## Data Availability

The datasets of the NIR spectroscopy and the LC-MS presented in this study are available in the research data repository of the University of Hamburg at: http://doi.org/10.25592/uhhfdm.16780 (accessed on 14 February 2025).

## Supplementary Materials

The following supporting information can be downloaded at: https://www.mdpi.com/article/10.3390/metabo15050330/s1, Figure S1: PCA score plot of the FT-NIR spectroscopy strawberry dataset of the MAP (M) and control (C) storage conditions, PCA score plot of the LC-MS dataset of the control storage and the MAP storage; Figure S2: MS/MS spectrum of the signal *m/z* 814.6292 at a retention time of 11.0 min in a strawberry sample extract. The collision energy was 20 eV. The compound was identified as PC 38:2; Figure S3: MS/MS spectrum of the signal *m/z* 601.5174 at a retention time of 14.6 min in a strawberry sample extract. The collision energy was 20 eV. The compound was identified as DG 36:3; Figure S4: MS/MS spectrum of the signal *m/z* 856.7013 at a retention time of 10.8 min in a strawberry sample extract. The collision energy was 20 eV. The compound was identified as 18:2 Glc sitosterol; Table S1: Metadata of the samples; Table S2: Overview of the liquid chromatography gradient. Both solvents contained an addition of 0.1 mMol/L ammonium formate; Table S3: Aerobic mesophilic bacteria count of both storages; Table S4: The counts of yeast and mold in both storage methods; Table S5: Water content [%] of intact strawberries measured using a dry scale; Table S6: ANOVA (*p*-value < 0.01) results for the FT-NIR dataset of the first 50 selected wavenumbers; Table S7: Summary of identification parameters of marker compounds that were selected by ANOVA (*p*-value < 0.01).

## References

[1] Kim D.-S., Park K.-J., Choi J.H., Lim J.-H., Kim H.-J. (2023). Metabolomic analysis of strawberries at different maturities according to postharvest storage period. Sci. Hortic..

[2] Giampieri F., Forbes-Hernandez T.Y., Gasparrini M., Alvarez-Suarez J.M., Afrin S., Bompadre S., Quiles J.L., Mezzetti B., Battino M. (2015). Strawberry as a health promoter: An evidence based review. Food Funct..

[3] Basu A., Nguyen A., Betts N.M., Lyons T.J. (2014). Strawberry as a functional food: An evidence-based review. Crit. Rev. Food Sci. Nutr..

[4] Drobek M., Cybulska J., Gałązka A., Feledyn-Szewczyk B., Marzec-Grządziel A., Sas-Paszt L., Gryta A., Trzciński P., Zdunek A., Frąc M. (2021). The use of interactions between microorganisms in strawberry cultivation (*Fragaria x ananassa* Duch.). Front. Plant Sci..

[5] Zhang H., Wang L., Dong Y., Jiang S., Cao J., Meng R. (2007). Postharvest biological control of gray mold decay of strawberry with *Rhodotorula glutinis*. Biol. Control.

[6] Allende A., Marín A., Buendía B., Tomás-Barberán F., Gil M.I. (2007). Impact of combined postharvest treatments (UV-C light, gaseous O_3_, superatmospheric O_2_ and high CO_2_) on health promoting compounds and shelf-life of strawberries. Postharvest Biol. Technol..

[7] Mahecha-Rubiano Y.R., Garavito J., Castellanos D.A. (2024). Configuration of a combined active packaging system with modified atmospheres and antimicrobial control to preserve fresh strawberry fruits. Food Packag. Shelf Life.

[8] Peano C., Giuggioli N.R., Girgenti V. (2014). Effect of different packaging materials on postharvest quality of cv. Envie2 strawberry. Int. Food Res. J..

[9] Sandhya (2010). Modified atmosphere packaging of fresh produce: Current status and future needs. LWT Food Sci. Technol..

[10] Abu-Zahra T.R. (2017). Effect of Cold Storage and Modified Atmosphere Packaging on Strawberry (*Fragaria X Ananassa* Duch.) cv.“Arben” Fruit Keeping Quality. Biosci. Biotechnol. Res. Asia.

[11] Liu Q., Wei K., Xiao H., Tu S., Sun K., Sun Y., Pan L., Tu K. (2019). Near-infrared hyperspectral imaging rapidly detects the decay of postharvest strawberry based on water-soluble sugar analysis. Food Anal. Methods.

[12] Shen F., Zhang B., Cao C., Jiang X. (2018). On-line discrimination of storage shelf-life and prediction of post-harvest quality for strawberry fruit by visible and near infrared spectroscopy. J. Food Process Eng..

[13] Mancini M., Mazzoni L., Gagliardi F., Balducci F., Duca D., Toscano G., Mezzetti B., Capocasa F. (2020). Application of the non-destructive NIR technique for the evaluation of strawberry fruits quality parameters. Foods.

[14] Šimkova K., Grohar M.C., Pelacci M., Veberic R., Jakopic J., Hudina M. (2024). The effect of freezing, frozen storage and thawing on the strawberry fruit composition. Int. J. Fruit Sci..

[15] Šimkova K., Veberič R., Hudina M., Jakopič J. (2024). Berry size and weight as factors influencing the chemical composition of strawberry fruit. J. Food Compos. Anal..

[16] Joshi P., Pahariya P., Al-Ani M.F., Choudhary R. (2022). Monitoring and prediction of sensory shelf-life in strawberry with ultraviolet-visible-near-infrared (UV-VIS-NIR) spectroscopy. Appl. Food Res..

[17] Saad A., Azam M.M., Amer B.M.A. (2022). Quality analysis prediction and discriminating strawberry maturity with a hand-held Vis–NIR spectrometer. Food Anal. Methods.

[18] Sánchez M.-T., de la Haba M.J., Benítez-López M., Fernández-Novales J., Garrido-Varo A., Pérez-Marín D. (2012). Non-destructive characterization and quality control of intact strawberries based on NIR spectral data. J. Food Eng..

[19] Giuggioli N.R., Girgenti V., Baudino C., Peano C. (2015). Influence of modified atmosphere packaging storage on postharvest quality and aroma compounds of strawberry fruits in a short distribution chain. J. Food Process. Preserv..

[20] Mawele Shamaila M., Powrie W.D., Skura B.J. (1992). Analysis of volatile compounds from strawberry fruit stored under modified atmosphere packaging (MAP). J. Food Sci..

[21] Shamaila M., Powrie W.D., Skura B.J. (1992). Sensory evaluation of strawberry fruit stored under modified atmosphere packaging (MAP) by quantitative descriptive analysis. J. Food Sci..

[22] Esmaeili Y., Zamindar N., Paidari S., Ibrahim S.A., Mohammadi Nafchi A. (2021). The synergistic effects of aloe vera gel and modified atmosphere packaging on the quality of strawberry fruit. J. Food Process. Preserv..

[23] Błaszczyk J., Bieniasz M., Nawrocki J., Kopeć M., Mierzwa-Hersztek M., Gondek K., Zaleski T., Knaga J., Bogdał S. (2022). The effect of harvest date and storage conditions on the quality of remontant strawberry cultivars grown in a gutter system under covers. Agriculture.

[24] Cheng Y.-T., Huang P.-H., Chan Y.-J., Chen S.-J., Lu W.-C., Li P.-H. (2023). A new strategy to design novel modified atmosphere packaging formulation maintains the qualities of postharvest strawberries (*Fragaria ananassa*) during low-temperature storage. J. Food Saf..

[25] Zhao X., Xia M., Wei X., Xu C., Luo Z., Mao L. (2019). Consolidated cold and modified atmosphere package system for fresh strawberry supply chains. LWT Food Sci. Technol..

[26] Olennikov D.N. (2022). Coumarins of lovage roots (*Levisticum officinale* WDJ Koch): LC-MS profile, quantification, and stability during postharvest storage. Metabolites.

[27] Pott D.M., de Abreu e Lima F., Soria C., Willmitzer L., Fernie A.R., Nikoloski Z., Osorio S., Vallarino J.G. (2020). Metabolic reconfiguration of strawberry physiology in response to postharvest practices. Food Chem..

[28] Van Meer G., Voelker D.R., Feigenson G.W. (2008). Membrane lipids: Where they are and how they behave. Nat. Rev. Mol. Cell Biol..

[29] Arndt M., Rurik M., Drees A., Ahlers C., Feldmann S., Kohlbacher O., Fischer M. (2021). Food authentication: Determination of the geographical origin of almonds (*Prunus dulcis* MILL.) via near-infrared spectroscopy. Microchem. J..

[30] Arndt M., Drees A., Ahlers C., Fischer M. (2020). Determination of the geographical origin of walnuts (*Juglans regia* L.) using near-infrared spectroscopy and chemometrics. Foods.

[31] Shakiba N., Gerdes A., Holz N., Wenck S., Bachmann R., Schneider T., Seifert S., Fischer M., Hackl T. (2022). Determination of the geographical origin of hazelnuts (*Corylus avellana* L.) by Near-Infrared spectroscopy (NIR) and a Low-Level Fusion with nuclear magnetic resonance (NMR). Microchem. J..

[32] Rinnan Å., van den Berg F., Engelsen S.B. (2009). Review of the most common pre-processing techniques for near-infrared spectra. TrAC Trends Anal. Chem..

[33] Geladi P., MacDougall D., Martens H. (1985). Linearization and scatter-correction for near-infrared reflectance spectra of meat. Appl. Spectrosc..

[34] Engel J., Gerretzen J., Szymańska E., Jansen J.J., Downey G., Blanchet L., Buydens L.M.C. (2013). Breaking with trends in pre-processing?. TrAC Trends Anal. Chem..

[35] Creydt M., Arndt M., Hudzik D., Fischer M. (2018). Plant metabolomics: Evaluation of different extraction parameters for nontargeted UPLC-ESI-QTOF-mass spectrometry at the example of white *Asparagus officinalis*. J. Agric. Food Chem..

[36] Bligh E.G., Dyer W.J. (1959). A rapid method of total lipid extraction and purification. Can. J. Biochem. Physiol..

[37] Sud M., Fahy E., Cotter D., Brown A., Dennis E.A., Glass C.K., Merrill A.H., Murphy R.C., Raetz C.R.H., Russell D.W. (2007). Lmsd: Lipid maps structure database. Nucleic Acids Res..

[38] Zhou Z., Tu J., Xiong X., Shen X., Zhu Z.-J. (2017). LipidCCS: Prediction of collision cross-section values for lipids with high precision to support ion mobility–mass spectrometry-based lipidomics. Anal. Chem..

[39] Pang Z., Lu Y., Zhou G., Hui F., Xu L., Viau C., Spigelman A.F., MacDonald P.E., Wishart D.S., Li S. (2024). MetaboAnalyst 6.0: Towards a unified platform for metabolomics data processing, analysis and interpretation. Nucleic Acids Res..

[40] Jalali A., Linke M., Geyer M., Mahajan P.V. (2020). Shelf life prediction model for strawberry based on respiration and transpiration processes. Food Packag. Shelf Life.

[41] Mastromatteo M., Mastromatteo M., Conte A., Del Nobile M.A. (2011). Combined effect of active coating and MAP to prolong the shelf life of minimally processed kiwifruit (*Actinidia deliciosa* cv. Hayward). Food Res. Int..

[42] Afifi E.H. (2016). Effect of active and passive modified atmosphere packaging on quality attributes of strawberry fruits during cold storage. Arab. Univ. J. Agric. Sci..

[43] Ozkaya O., Dündar O., Scovazzo G.C., Volpe G. (2009). Evaluation of quality parameters of strawberry fruits in modified atmosphere packaging during storage. Afr. J. Biotechnol..

[44] (2005). No. 2073/2005 of 15 November 2005 on Microbiological Criteria for Foodstuffs.

[45] Wang W., Hu W., Ding T., Ye X., Liu D. (2018). Shelf-life prediction of strawberry at different temperatures during storage using kinetic analysis and model development. J. Food Process. Preserv..

[46] Siro I., Devlieghere F., Jacxsens L., Uyttendaele M., Debevere J. (2006). The microbial safety of strawberry and raspberry fruits packaged in high-oxygen and equilibrium-modified atmospheres compared to air storage. Int. J. Food..

[47] de Filho M.J., Scolforo C.Z., Saraiva S.H., Pinheiro C.J.G., Silva P.I., Della Lucia S.M. (2018). Physicochemical, microbiological and sensory acceptance alterations of strawberries caused by gamma radiation and storage time. Sci. Hortic..

[48] Ladika G., Strati I.F., Tsiaka T., Cavouras D., Sinanoglou V.J. (2024). On the assessment of strawberries’ shelf-life and quality, based on image analysis, physicochemical methods, and chemometrics. Foods.

[49] Pelletier W., Brecht J.K., do Nascimento Nunes M.C., Emond J.-P. (2011). Quality of strawberries shipped by truck from California to Florida as influenced by postharvest temperature management practices. HortTechnology.

[50] Giampieri F., Tulipani S., Alvarez-Suarez J.M., Quiles J.L., Mezzetti B., Battino M. (2012). The strawberry: Composition, nutritional quality, and impact on human health. Nutrition.

[51] Workman J., Weyer L. (2012). Practical Guide and Spectral Atlas for Interpretive Near-Infrared.

[52] Amodio M.L., Ceglie F., Chaudhry M.M.A., Piazzolla F., Colelli G. (2017). Potential of NIR spectroscopy for predicting internal quality and discriminating among strawberry fruits from different production systems. Postharvest Biol. Technol..

[53] Mangaraj S., Goswami T.K. (2011). Measurement and modeling of respiration rate of guava (CV. *Baruipur*) for modified atmosphere packaging. Int. J. Food Prop..

[54] Todeschini V., AitLahmidi N., Mazzucco E., Marsano F., Gosetti F., Robotti E., Bona E., Massa N., Bonneau L., Marengo E. (2018). Impact of beneficial microorganisms on strawberry growth, fruit production, nutritional quality, and volatilome. Front. Plant Sci..

[55] Badillo G.M., Segura-Ponce L.A. (2020). Classic and reaction-diffusion models used in modified atmosphere packaging (MAP) of fruit and vegetables. Food Eng. Rev..

[56] Mangaraj S., Goswami T.K. (2011). Modeling of respiration rate of litchi fruit under aerobic conditions. Food Bioprocess Technol..

[57] Cybulska J., Drobek M., Panek J., Cruz-Rubio J.M., Kurzyna-Szklarek M., Zdunek A., Frąc M. (2022). Changes of pectin structure and microbial community composition in strawberry fruit (*Fragaria × ananassa* Duch.) during cold storage. Food Chem..

[58] Frick A.A., Weyermann C. (2019). An untargeted lipidomic approach for qualitative determination of latent fingermark glycerides using UPLC-IMS-QToF-MS E. Analyst.

[59] Pi J., Wu X., Feng Y. (2016). Fragmentation patterns of five types of phospholipids by ultra-high-performance liquid chromatography electrospray ionization quadrupole time-of-flight tandem mass spectrometry. Anal. Methods.

[60] Song C., Wang K., Xiao X., Liu Q., Yang M., Li X., Feng Y., Li S., Shi L., Chen W. (2022). Membrane lipid metabolism influences chilling injury during cold storage of peach fruit. Food Res. Int..

[61] Hong K., Yao Q., Golding J.B., Pristijono P., Zhang X., Hou X., Yuan D., Li Y., Chen L., Song K. (2023). Low temperature storage alleviates internal browning of ‘*Comte de Paris*’ winter pineapple fruit by reducing phospholipid degradation, phosphatidic acid accumulation and membrane lipid peroxidation processes. Food Chem..

[62] Aktas M., Wessel M., Hacker S., Klüsener S., Gleichenhagen J., Narberhaus F. (2010). Phosphatidylcholine biosynthesis and its significance in bacteria interacting with eukaryotic cells. Eur. J. Cell Biol..

[63] Sohlenkamp C., López-Lara I.M., Geiger O. (2003). Biosynthesis of phosphatidylcholine in bacteria. Prog. Lipid Res..

[64] Wang X. (2002). Phospholipase D in hormonal and stress signaling. Curr. Opin. Plant Biol..

[65] Ali U., Lu S., Fadlalla T., Iqbal S., Yue H., Yang B., Hong Y., Wang X., Guo L. (2022). The functions of phospholipases and their hydrolysis products in plant growth, development and stress responses. Prog. Lipid Res..

[66] Zhou Y., Pan X., Qu H., Underhill S. (2014). Low temperature alters plasma membrane lipid composition and ATPase activity of pineapple fruit during blackheart development. J. Bioenerg. Biomembr..

[67] Whitaker B.D. (1991). Changes in lipids of tomato fruit stored at chilling and non-chilling temperatures. Phytochemistry.

[68] Bartley I.M. (1986). Changes in sterol and phospholipid composition of apples during storage at low temperature and low oxygen concentration. J. Sci. Food Agric..

[69] Lee S., Suh S., Kim S., Crain R.C., Kwak J.M., Nam H.-G., Lee Y. (1997). Systemic elevation of phosphatidic acid and lysophospholipid levels in wounded plants. Plant J..

[70] Narváez-Vásquez J., Florin-Christensen J., Ryan C.A. (1999). Positional specificity of a phospholipase a activity induced by wounding, systemin, and oligosaccharide elicitors in tomato leaves. Plant Cell.

[71] Drobek M., Frąc M., Zdunek A., Cybulska J. (2020). The effect of cultivation method of strawberry (*Fragaria x ananassa* Duch.) cv. Honeoye on structure and degradation dynamics of pectin during cold storage. Molecules.

[72] Saint Angelo A.J., Vercellotti J., Jacks T., Legendre M. (1996). Lipid oxidation in foods. Crit. Rev. Food Sci. Nutr..

[73] Loesel H., Shakiba N., Wenck S., Le Tan P., Karstens T.-O., Creydt M., Seifert S., Hackl T., Fischer M. (2023). Food monitoring: Limitations of accelerated storage to predict molecular changes in hazelnuts (*Corylus avellana* L.) under Realistic conditions using UPLC-ESI-IM-QTOF-MS. Metabolites.

[74] Gülen H., Çetinkaya C., Kadıoğlu M., Kesici M., Cansev A., Eriş A. (2008). Peroxidase activity and lipid peroxidation in strawberry (*Fragaria x ananassa*) plants under low temperature. J. Biol. Environ. Sci..

