# Supplementary material for: Storage Profiling: Evaluating the Effect of Modified Atmosphere Packaging on Metabolomic Changes of Strawberries (Fragaria × ananassa)
Source: Metabolites. 2025 May 15;15(5):330. doi: 10.3390/metabo15050330 (PMC12113444; doi:10.3390/metabo15050330)
Supplement: Supplementary file 1 [file metabolites-15-00330-s001.zip › metabolites-3637365-supplementary.pdf]

Electronic Supplementary Information

Storage Profiling: Evaluating the Effect of Modified Atmosphere Packaging on Metabolomic Changes of Strawberries (*Fragaria × ananassa*)

Johannes Brockelt[1], Robin Dammann[1], Jennifer Griese[2], Agnes Weiss[2], Markus Fischer[1], Marina Creydt[1,*]

[1] Hamburg School of Food Science, Institute of Food Chemistry, University of Hamburg, Grindelallee 117, 20146 Hamburg, Germany

[2] Hamburg School of Food Science, Food Microbiology, University of Hamburg, Ohnhorststrasse 18, 22609 Hamburg, Germany

*Corresponding author:

Marina Creydt, Hamburg School of Food Science - Institute of Food Chemistry, University of Hamburg, Grindelallee 117, 20146 Hamburg, Germany

Tel.: +49 40 42838-8803, Fax: +49 40 42838-4342

Email: marina.creydt@uni-hamburg.de

Table S1: Metadata of the samples.

Storage Condition	Sample ID	Storage Day	Storage Condition	Sample ID	Storage Day
Control	C0-1	CDay0	MAP	C0-1	CDay0
Control	C0-2	CDay0	MAP	C0-2	CDay0
Control	C0-3	CDay0	MAP	C0-3	CDay0
Control	C0-4	CDay0	MAP	C0-4	CDay0
Control	C0-5	CDay0	MAP	C0-5	CDay0
Control	C0-6	CDay0	MAP	C0-6	CDay0
Control	C0-7	CDay0	MAP	C0-7	CDay0
Control	C0-8	CDay0	MAP	C0-8	CDay0
Control	C0-9	CDay0	MAP	C0-9	CDay0
Control	C02-1	CDay2	MAP	M02-1	MDay2
Control	C02-2	CDay2	MAP	M02-2	MDay2
Control	C02-3	CDay2	MAP	M02-3	MDay2
Control	C02-4	CDay2	MAP	M02-4	MDay2
Control	C02-5	CDay2	MAP	M02-5	MDay2
Control	C02-6	CDay2	MAP	M02-6	MDay2
Control	C02-7	CDay2	MAP	M02-7	MDay2
Control	C02-8	CDay2	MAP	M02-8	MDay2
Control	C02-9	CDay2	MAP	M02-9	MDay2
Control	C04-1	CDay4	MAP	M04-1	MDay4
Control	C04-2	CDay4	MAP	M04-2	MDay4
Control	C04-3	CDay4	MAP	M04-3	MDay4
Control	C04-4	CDay4	MAP	M04-4	MDay4
Control	C04-5	CDay4	MAP	M04-5	MDay4
Control	C04-6	CDay4	MAP	M04-6	MDay4
Control	C04-7	CDay4	MAP	M04-7	MDay4
Control	C04-8	CDay4	MAP	M04-8	MDay4
Control	C04-9	CDay4	MAP	M04-9	MDay4
Control	C06-1	CDay6	MAP	M06-1	MDay6
Control	C06-2	CDay6	MAP	M06-2	MDay6
Control	C06-3	CDay6	MAP	M06-3	MDay6
Control	C06-4	CDay6	MAP	M06-4	MDay6
Control	C06-5	CDay6	MAP	M06-5	MDay6
Control	C06-6	CDay6	MAP	M06-6	MDay6
Control	C06-7	CDay6	MAP	M06-7	MDay6
Control	C06-8	CDay6	MAP	M06-8	MDay6
Control	C06-9	CDay6	MAP	M06-9	MDay6
Control	C08-1	CDay8	MAP	M08-1	MDay8
Control	C08-2	CDay8	MAP	M08-2	MDay8
Control	C08-3	CDay8	MAP	M08-3	MDay8
Control	C08-4	CDay8	MAP	M08-4	MDay8
Control	C08-5	CDay8	MAP	M08-5	MDay8
Control	C08-6	CDay8	MAP	M08-6	MDay8
Control	C08-7	CDay8	MAP	M08-7	MDay8
Control	C08-8	CDay8	MAP	M08-8	MDay8
Control	C08-9	CDay8	MAP	M08-9	MDay8
Control	C10-1	CDay10	MAP	M10-1	MDay10
Control	C10-2	CDay10	MAP	M10-2	MDay10
Control	C10-3	CDay10	MAP	M10-3	MDay10
Control	C10-4	CDay10	MAP	M10-4	MDay10

Storage Condition	Sample ID	Storage Day	Storage Condition	Sample ID	Storage Day
Control	C10-5	CDay10	MAP	M10-5	MDay10
Control	C10-6	CDay10	MAP	M10-6	MDay10
Control	C10-7	CDay10	MAP	M10-7	MDay10
Control	C10-8	CDay10	MAP	M10-8	MDay10
Control	C10-9	CDay10	MAP	M10-9	MDay10

Table S2: Overview of the liquid chromatography gradient. Both solvents contained an addition of 0.1 mMol/L ammonium formate.

Time [min]	Water [%]	Isopropanol/Acetonitrile (2:1, v/v) [%]
0	35	65
2	35	65
4	15	85
22	0	100
23	0	100
25	35	65
30	35	65

Table S3: Aerobic mesophilic bacteria count of both storages.

Aerobic mesophilic bacteria count									
**Storage condition**	**Day**	**Viable count [CFU/g]**	**Average viable count [CFU/g]**	**Standard deviation**	**Storage condition**	**Day**	**Viable count [CFU/g]**	**Average viable count [CFU/g]**	**Standard deviation**
Control	0	3.5	3.5	0.0	Control	0	3.5	3.5	0.0
Control	0	3.5			Control	0	3.5		
Control	0	3.5			Control	0	3.5		
Control	2	3.8	3.8	0.1	MAP	2	3.5	3.5	0.1
Control	2	3.8			MAP	2	3.6		
Control	2	3.7			MAP	2	3.5		
Control	4	4.0	4.0	0.0	MAP	4	3.6	3.6	0.1
Control	4	4.0			MAP	4	3.7		
Control	4	4.0			MAP	4	3.6		
Control	6	4.2	4.2	0.0	MAP	6	3.7	3.8	0.1
Control	6	4.2			MAP	6	3.8		
Control	6	4.2			MAP	6	3.8		
Control	8	4.6	4.6	0.0	MAP	8	4.3	4.3	0.1
Control	8	4.6			MAP	8	4.3		
Control	8	4.6			MAP	8	4.2		
Control	10	5.0	5.0	0.0	MAP	10	4.6	4.6	0.0
Control	10	5.0			MAP	10	4.6		
Control	10	5.0			MAP	10	4.6		

Table S4: The counts of yeast and mold in both storage methods.

**Yeast and mold count**									
**Storage condition**	**Day**	**Viable count [CFU/g]**	**Average viable count [CFU/g]**	**Standard deviation**		**Day**	**Viable count [CFU/g]**	**Average viable count [CFU/g]**	**Standard deviation**
Control	0	3.3	3.4	0.1	Control	0	3.3	3.4	0.1
Control	0	3.3			Control	0	3.3		
Control	0	3.4			Control	0	3.4		
Control	2	3.5	3.6	0.1	MAP	2	3.5	3.4	0.1
Control	2	3.6			MAP	2	3.4		
Control	2	3.6			MAP	2	3.4		
Control	4	3.9	3.9	0.0	MAP	4	3.4	3.5	0.1
Control	4	3.9			MAP	4	3.5		
Control	4	3.9			MAP	4	3.4		
Control	6	4.1	4.1	0.0	MAP	6	3.7	3.7	0.1
Control	6	4.1			MAP	6	3.7		
Control	6	4.1			MAP	6	3.6		
Control	8	4.5	4.5	0.0	MAP	8	4.1	4.1	0.1
Control	8	4.5			MAP	8	4.1		
Control	8	4.5			MAP	8	4.0		
Control	10	4.8	4.8	0.1	MAP	10	4.4	4.4	0.1
Control	10	4.7			MAP	10	4.4		
Control	10	4.8			MAP	10	4.5		

Table S5: Water content [%] of intact strawberries measured using a dry scale.

Storage condition	Day	Water content [%]	Average [%]	Standard deviation	Storage condition	Day	Water content [%]	Average [%]	Standard deviation
Control	0	90.64	90.24	0.31	Control	0	90.64	90.24	0.31
Control	0	90.72			Control	0	90.72		
Control	0	89.96			Control	0	89.96		
Control	0	90.02			Control	0	90.02		
Control	0	89.76			Control	0	89.76		
Control	0	90.32			Control	0	90.32		
Control	0	89.97			Control	0	89.97		
Control	0	90.46			Control	0	90.46		
Control	0	90.31			Control	0	90.31		
Control	2	89.23	89.26	0.39	MAP	2	90.75	90.09	0.38
Control	2	89.43			MAP	2	89.67		
Control	2	89.32			MAP	2	89.89		
Control	2	88.67			MAP	2	89.76		
Control	2	89.39			MAP	2	90.67		
Control	2	88.65			MAP	2	89.77		
Control	2	89.78			MAP	2	89.66		
Control	2	89.05			MAP	2	90.33		
Control	2	89.78			MAP	2	90.29		
Control	4	88.04	87.69	0.41	MAP	4	89.77	89.79	0.38
Control	4	87.39			MAP	4	89.45		
Control	4	87.46			MAP	4	90.14		
Control	4	87.46			MAP	4	90.26		
Control	4	88.12			MAP	4	89.12		
Control	4	87.01			MAP	4	90.05		
Control	4	87.46			MAP	4	89.55		
Control	4	87.89			MAP	4	90.19		
Control	4	88.34			MAP	4	90.2		
Control	6	85.87	86.27	0.32	MAP	6	89.56	89.03	0.46
Control	6	86.43			MAP	6	89.44		
Control	6	86.37			MAP	6	89.13		
Control	6	85.77			MAP	6	88.56		
Control	6	86.63			MAP	6	88.32		
Control	6	86.66			MAP	6	89.45		
Control	6	86.26			MAP	6	88.96		
Control	6	85.94			MAP	6	88.44		
Control	6	86.54			MAP	6	89.45		
Control	8	84.33	84.10	0.42	MAP	8	89.03	88.80	0.32
Control	8	84.04			MAP	8	88.99		
Control	8	83.83			MAP	8	88.09		
Control	8	84.54			MAP	8	88.49		
Control	8	83.43			MAP	8	88.89		
Control	8	84.08			MAP	8	89.10		
Control	8	84.73			MAP	8	88.78		
Control	8	83.53			MAP	8	88.69		
Control	8	84.41			MAP	8	89.15		
Control	10	83.72	83.72	0.35	MAP	10	88.09	87.97	0.30
Control	10	84.08			MAP	10	88.14		
Control	10	83.42			MAP	10	87.71		

Storage condition	Day	Water content [%]	Average [%]	Standard deviation	Storage condition	Day	Water content [%]	Average [%]	Standard deviation
Control	10	83.03			MAP	10	88.11		
Control	10	84.21			MAP	10	87.75		
Control	10	83.56			MAP	10	88.32		
Control	10	83.88			MAP	10	88.42		
Control	10	84.01			MAP	10	87.45		
Control	10	83.56			MAP	10	87.78		

Table S6: ANOVA (*p*-value < 0.01) results for the FT-NIR data set of the first 50 selected wavenumbers.

Number	Wavenumber [cm^{-1}]	*p*-values
1	5,340.816	1.305e-28
2	5,351.02	1.7794e-26
3	8,800	1.5789e-25
4	8,789.796	3.5794e-25
5	5,330.612	2.21e-24
6	8,779.592	3.0061e-24
7	8,810.204	4.8931e-24
8	4,504.082	9.1596e-24
9	4,493.877	1.6039e-23
10	4,514.286	2.984e-23
11	8,769.388	4.8437e-23
12	4,524.49	1.1233e-22
13	4,483.673	1.4983e-22
14	5,361.224	3.6689e-22
15	10,330.61	4.6512e-22
16	10,269.39	6.4356e-22
17	8,759.184	8.2372e-22
18	4,534.694	1.1767e-21
19	10,300	1.5083e-21
20	10,310.2	2.1745e-21
21	10,289.8	2.4326e-21
22	10,351.02	2.8731e-21
23	4,473.469	3.9029e-21

Number	Wavenumber [cm^{-1}]	*p*-values
24	10,361.22	3.9201e-21
25	10,320.41	3.9593e-21
26	10,340.82	4.8975e-21
27	8,748.979	5.1018e-21
28	10,279.59	6.4198e-21
29	10,259.18	8.0038e-21
30	8,820.408	9.8013e-21
31	8,738.775	1.2536e-20
32	4,544.898	1.75e-20
33	7,126.53	2.4739e-20
34	10,371.43	3.2026e-20
35	7,116.326	3.2616e-20
36	7,136.734	3.3734e-20
37	10,248.98	3.8576e-20
38	10,402.04	4.5494e-20
39	8,728.571	7.0897e-20
40	7,106.122	8.4482e-20
41	7,146.938	8.7563e-20
42	10,381.63	1.3054e-19
43	10,391.84	1.8536e-19
44	10,238.78	2.5293e-19
45	10,412.24	2.5328e-19
46	8,718.367	2.8089e-19
47	4,555.102	3.0164e-19
48	7,095.918	3.3399e-19
49	7,157.143	4.1296e-19
50	4,463.265	4.9014e-19

Table S7: Summary of identification parameters of marker compounds that were selected by ANOVA (*p*-value < 0.01).

Tentative Metabolite	*p*-value	FDR	Sum formula	Ion	*m/z* (analytical)	*m/z* (calculated)	Δ *m/z* [Da]	RT [min]	DT [ms]	CCS-value (analytical) [Å²]	CCS-value (LipidCCS database) [Å²]	Main fragments [*m/z*]
PC (30:2)	9.05e-20	3.40e-18	$C_{38}H_{72}NO_8P$	H+		702.5068	0.0025	7.3	36.10	276.53	276.2	184.07
PC (32:0)	1.15e-40	6.67e-38	$C_{40}H_{80}NO_8P$	H+	734.5671	734.5694	0.0023	9.6	38.12	283.42	282.1-290.2	184.07
PC (32:1)	2.93e-31	2.99e-31	$C_{40}H_{78}NO_8P$	H+	732.5538	732.5511	0.0027	8.3	37.79	280.99	272.7-284.7	184.07
PC (33:1)	4.56e-15	1.00e-13	$C_{41}H_{80}NO_8P$	H+	746.5664	746.5694	0.0030	9.0	38.16	288.66	288.0	184.07
PC (33:2)	3.03e-27	2.51e-25	$C_{41}H_{78}NO_8P$	H+	744.5518	744.5538	0.0020	8.5	37.64	279.78	269.4-285.1	184.07
PC (33:3)	4.65e-32	8.22e-32	$C_{41}H_{76}NO_8P$	H+	742.5365	742.5381	0.0016	7.7	37.08	275.55	275.2	184.07
PC (34:1)	1.43e-22	7.18e-21	$C_{42}H_{82}NO_8P$	H+	760.5836	760.5851	0.0015	9.6	38.56	286.48	289.5	184.04
PC (34:3)	6.81e-17	1.95e-15	$C_{42}H_{78}NO_8P$	H+	756.5528	756.5538	0.0010	7.7	37.88	281.45	270.8-284.1	184.07
PC (35:2)	2.09e-19	7.54e-18	$C_{43}H_{82}NO_8P$	H+	772.5832	772.5851	0.0019	9.2	38.65	287.11	289.8	184.07
PC (35:5)	1.21e-40	6.66e-38	$C_{43}H_{76}NO_8P$	H+	766.5329	766.5381	0.0052	8.5	38.30	284.56	284.4	184.07
PC (36:1)	1.19e-33	2.46e-31	$C_{44}H_{86}NO_8P$	H+	788.6136	788.6164	0.0028	11.1	39.36	292.29	277.9-293.9	184.07
PC (36:2)	3.46e-48	5.73e-45	$C_{44}H_{84}NO_8P$	H+	786.5995	786.6007	0.0012	9.7	39.13	290.61	290.5	184.07
PC (36:3)	3.31e-35	9.13e-33	$C_{44}H_{82}NO_8P$	H+	784.5823	784.5851	0.0058	8.4	38.98	289.49	290.0	184.07
PC (37:2)	3.00e-21	1.30e-19	$C_{45}H_{86}NO_8P$	H+	800.6142	800.6164	0.0022	10.4	39.33	291.98	281.9-294.3	184.07
PC (37:2)	3.45e-17	1.06e-15	$C_{45}H_{86}NO_8P$	NH_4+	817.6299	817.6429	0.0130	11.3	40.10	294.61	294.4	184.07
PC (38:2)	1.36e-28	1.32e-26	$C_{46}H_{88}NO_8P$	H+	814.6292	814.6320	0.0028	11.0	39.83	295.63	295.1	184.07
PC (38:4)	2.61e-29	2.88e-27	$C_{46}H_{84}NO_8P$	H+	810.6007	810.5954	0.0053	11.0	39.69	294.63	294.6	184.07
PC (38:5)	1.34e-33	2.46e-31	$C_{46}H_{82}NO_8P$	H+	808.5810	808.5851	0.0041	9.6	39.54	293.52	293.4	184.07
PC (38:6)	1.66e-35	5.50e-33	$C_{46}H_{80}NO_8P$	H+	806.5659	806.5694	0.0035	8.5	39.42	292.65	292.6	184.07
PC (38:7)	2.84e-18	9.24e-17	$C_{46}H_{78}NO_8P$	H+	804.5482	804.5538	0.0056	6.1	38.94	289.03	285.5-290.5	184.07
PC (40:5)	1.88e-24	1.15e-22	$C_{48}H_{86}NO_8P$	H+	836.6112	836.6164	0.0052	11.0	40.21	298.37	298.1	184.07
LPC (16:0)	3.17e-22	1.29e-26	$C_{24}H_{50}NO_7P$	H+	496.3378	496.3398	0.0020	3.4	30.55	228.87	227.0-232.3	184.07
LPC (18:0)	1.25e-28	1.29e-26	$C_{26}H_{54}NO_7P$	H+	524.3711	524.3692	0.0019	4.5	31.61	238.53	238.5-240.7	184.07
LPC (18:1)	1.57e-24	1.04e-22	$C_{26}H_{52}NO_7P$	H+	522.3554	522.3533	0.0021	3.6	30.95	236.2	313.8	184.07
LPC (18:2)	3.22e-19	1.13e-17	$C_{26}H_{50}NO_7P$	H+	520.3398	520.3378	0.0020	2.8	30.28	233.6	316.1	184.07
LPC (18:3)	6.72e-24	3.84e-22	$C_{26}H_{48}NO_7P$	H+	518.3241	518.3196	0.0045	3.4	30.92	231.9	320.1	184.07
PC-O (34:2)	1.80e-16	4.97e-15	$C_{42}H_{80}NO_7P$	H+	774.5613	774.5643	0.0030	6.1	38.54	286.28	no entry	184.07
PC-O (38:0)	3.16e-21	1.34e-19	$C_{46}H_{94}NO_7P$	H+	786.6570	786.6735	0.0165	11.4	39.34	291.13	291.1	184.07
DG (34:1)	7.69e-26	6.07e-24	$C_{37}H_{70}O_5$	H-H_2O+	577.5173	577.5173	0.0017	4.6	34.50	262.67	262.5	265.25
DG (36:3)	7.42e-19	8.38e-19	$C_{39}H_{70}O_5$	H-H_2O+	601.5174	601.5196	0.0022	14.6	34.55	257.76	255.9-265.4	265.25
DG (36:4)	2.35e-14	2.34e-14	$C_{39}H_{68}O_5$	H-H_2O+	599.5014	599.5034	0.0020	7.1	34.21	258.24	258.9	263.23
18:2-Glc-sitosterol	3.22e-15	1.58e-14	$C_{53}H_{90}O_7$	Na+	861.6579	861.6576	0.0003	10.8	44.76	293.8	no entry	261.22; 335.26; 397.38
18:2-Glc-sitosterol	3.22e-15	1.12e-19	$C_{53}H_{90}O_7$	NH_4+	856.7013	856.7025	0.0012	10.8	45.08	292.88	no entry	397.38; 425.29

Tentative Metabolite	*p*-value	FDR	Sum formula	Ion	*m/z* (analytical)	*m/z* (calculated)	Δ *m/z* [Da]	RT [min]	DT [ms]	CCS-value (analytical) [Å^2]	CCS-value (LipidCCS database) [Å^2]	Main fragments [*m/z*]
18:2-Glc-stigmasterol	1.69e-23	9.03e-22	$C_{53}H_{88}O_7$	NH_4+	854.6856	854.6868	0.0012	10.0	44.70	331.68	no entry	395.37

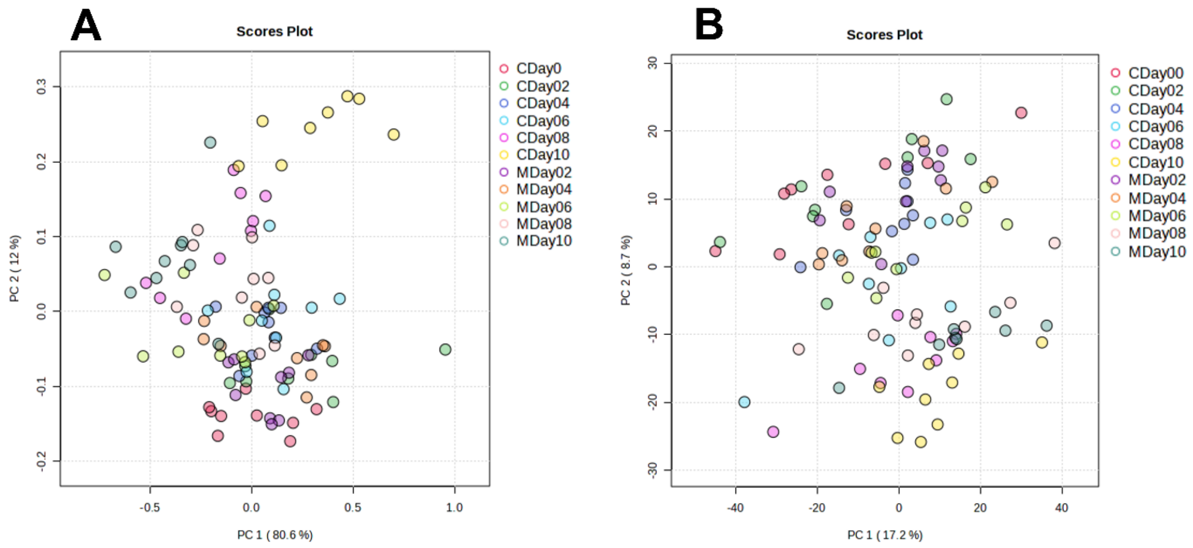

Figure S1: PCA score plot of the FT-NIR spectroscopy strawberry dataset of the MAP (M) and control (C) storage conditions (Figure S1A). PCA score plot of the LC-MS dataset of the control storage and the MAP storage (Figure S1B).

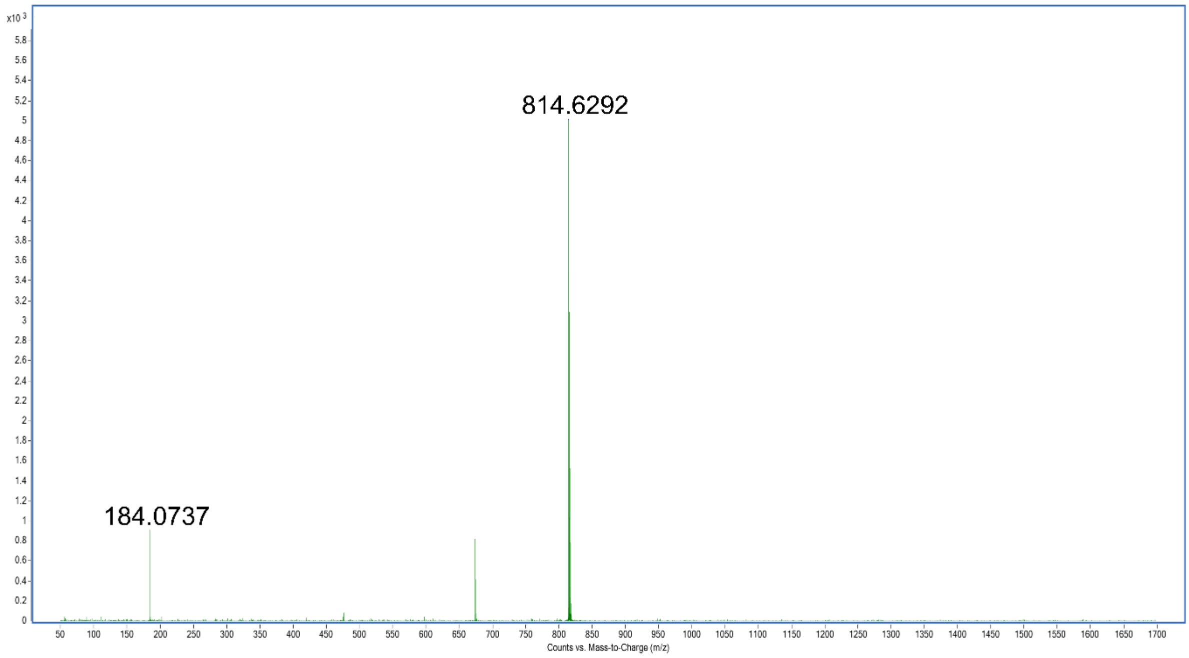

Figure S2: MS/MS spectrum of the signal *m/z* 814.6292 at a retention time of 11.0 min in a strawberry sample extract. The collision energy was 20 eV. The compound was identified as PC 38:2.

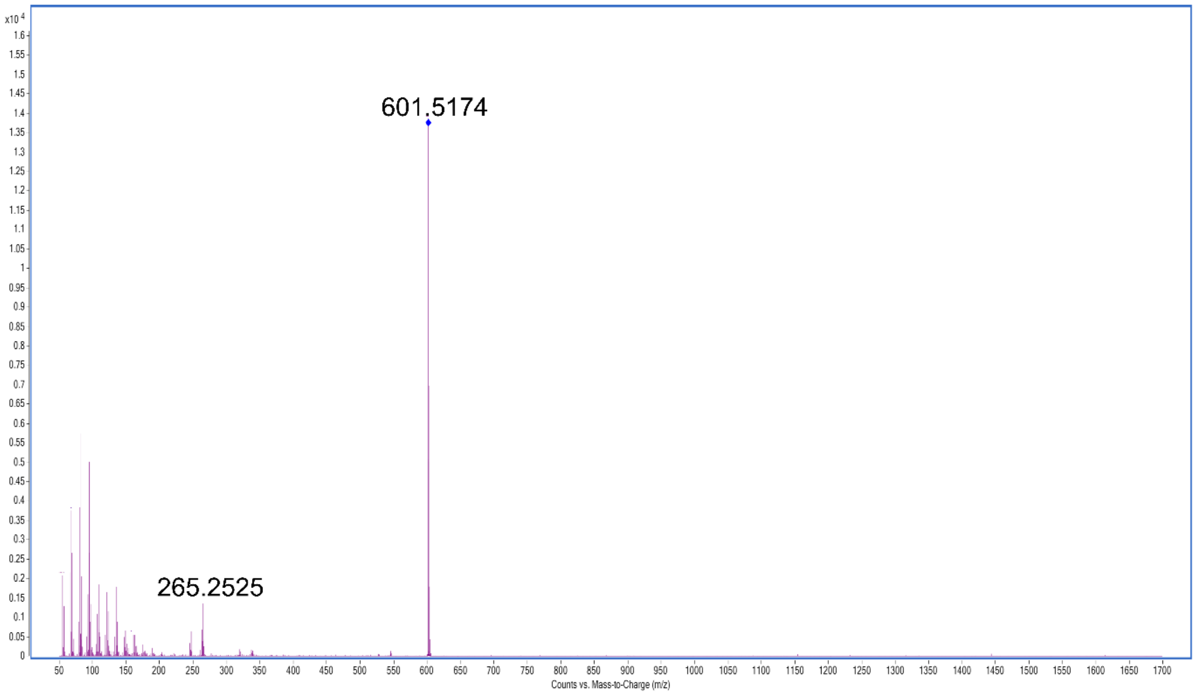

Figure S3: MS/MS spectrum of the signal *m*/*z* 601.5174 at a retention time of 14.6 min in a strawberry sample extract. The collision energy was 20 eV. The compound was identified as DG 36:3.

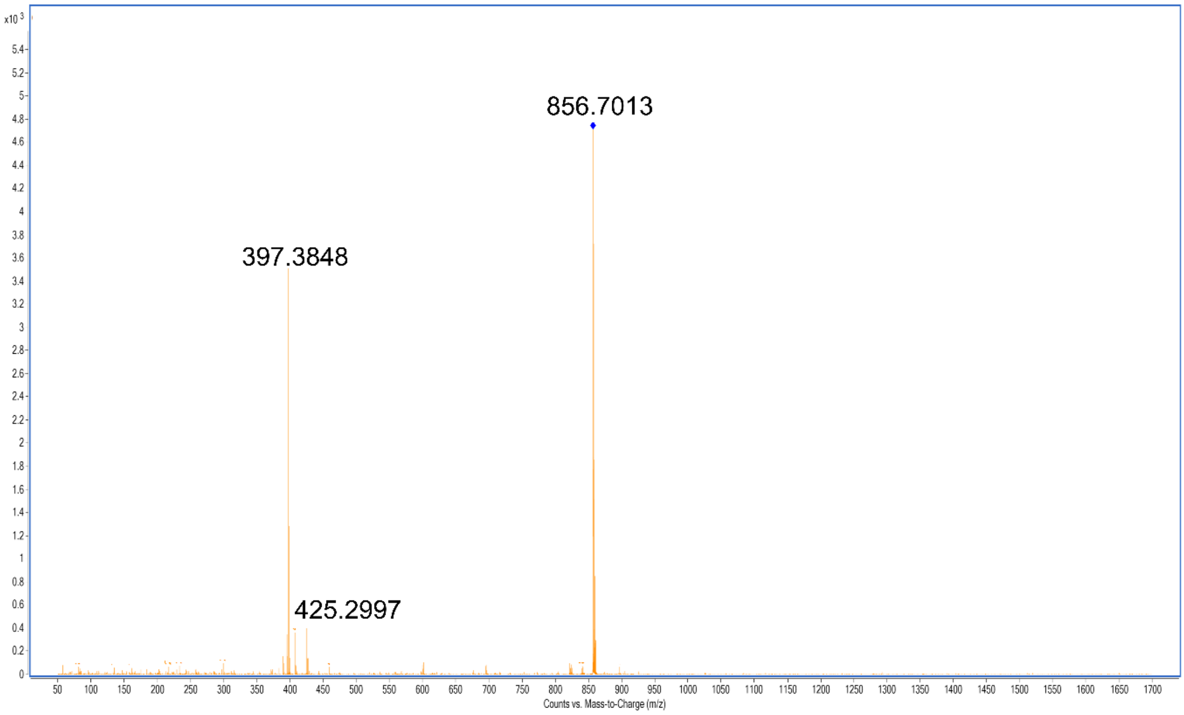

Figure S4: MS/MS spectrum of the signal *m*/*z* 856.7013 at a retention time of 10.8 min in a strawberry sample extract. The collision energy was 20 eV. The compound was identified as 18:2-Glc-sitosterol.
